# Buyang Huanwu Decoction promotes neurorepair after spinal cord injury through a *Lactobacillus johnsonii*–indole-3-lactic acid–AhR–PI3K/Akt axis

**DOI:** 10.1186/s13020-026-01408-x

**Published:** 2026-05-08

**Authors:** Jinwang Dong, Yang Cao, Xiujin Chen, Tao Xie, Xiaobo Zhang, Qingpeng Zhao, Cunhu Shi, Qiangqiang Miao, Zhengwei Xu, Liang Yan, Liang Dong

**Affiliations:** 1 Department of Spine Surgery, Honghui Hospital, Xi’an Jiaotong University, Xi’an, Shaanxi China; 2 Medical College of Yan’n University, Yan’an University, Yan’an, Shaanxi China; 3 Department of Orthopedic Oncology, Honghui Hospital, Xi’an Jiaotong University, Xi’an, Shaanxi China; 4 Xi’an Medical Emergency Center, Xi’an, Shaanxi China; 5 Department of Spine Surgery, Shenzhen Pingle Orthopedic Hospital (Shenzhen Pingshan District Traditional Chinese Medicine Hospital), Shenzhen, 518118 Guangdong People’s Republic of China

**Keywords:** Spinal cord injury, Buyang Huanwu Decoction, Fecal microbiota transplantation, Gut–Spinal cord axis, *Lactobacillus johnsonii*, Indole-3-lactic acid

## Abstract

**Background:**

Spinal cord injury (SCI) induces gut microbiota dysbiosis, which significantly affects recovery. Buyang Huanwu Decoction (BHD), a traditional Chinese medicine formula, has shown therapeutic effects on SCI. Although BHD is known to modulate gut microbiota, whether its benefits are mediated through the gut–spinal cord axis remains unclear.

**Methods:**

A rat SCI model was established. BHD was administered orally, and fecal microbiota transplantation (FMT) from BHD-treated rats (BHD-FMT) was performed to assess neuroprotective and gut-protective effects. Behavioral testing, histology, and immunofluorescence evaluated motor recovery, inflammation, and neuroregeneration. Gut microbiota profiling was performed using 16S rDNA sequencing and metagenomics, while targeted metabolomics quantified tryptophan metabolites. Transcriptomics validated key pathways, and a microbiota–metabolite–signaling network was constructed.

**Results:**

BHD significantly improved motor function, reduced spinal inflammation, and promoted neuronal survival and axonal regeneration. It restored gut function, reduced colonic inflammation, and enhanced ZO-1 and Occludin expression, which were further confirmed by FMT. BHD-FMT reshaped the gut microbiota and enriched *Lactobacillus johnsonii*, which correlated positively with recovery. Metabolomics showed increased tryptophan metabolites, including indole-3-lactic acid (ILA) and indole-3-propionic acid (IPA), with ILA strongly associated with functional improvement. Transcriptomic analysis and Western blot validation demonstrated that BHD-FMT activated the AhR–PI3K/Akt pathway, which was suppressed by an AhR antagonist.

**Conclusion:**

BHD promotes neuroregeneration after SCI by reshaping gut microbiota and enhancing tryptophan metabolism, potentially exerting its effects through the *L. johnsonii*–ILA–AhR–PI3K/Akt network. These findings reveal a gut–spinal cord axis–mediated mechanism of BHD and highlight microecological targets for SCI therapy.

**Graphical Abstract:**

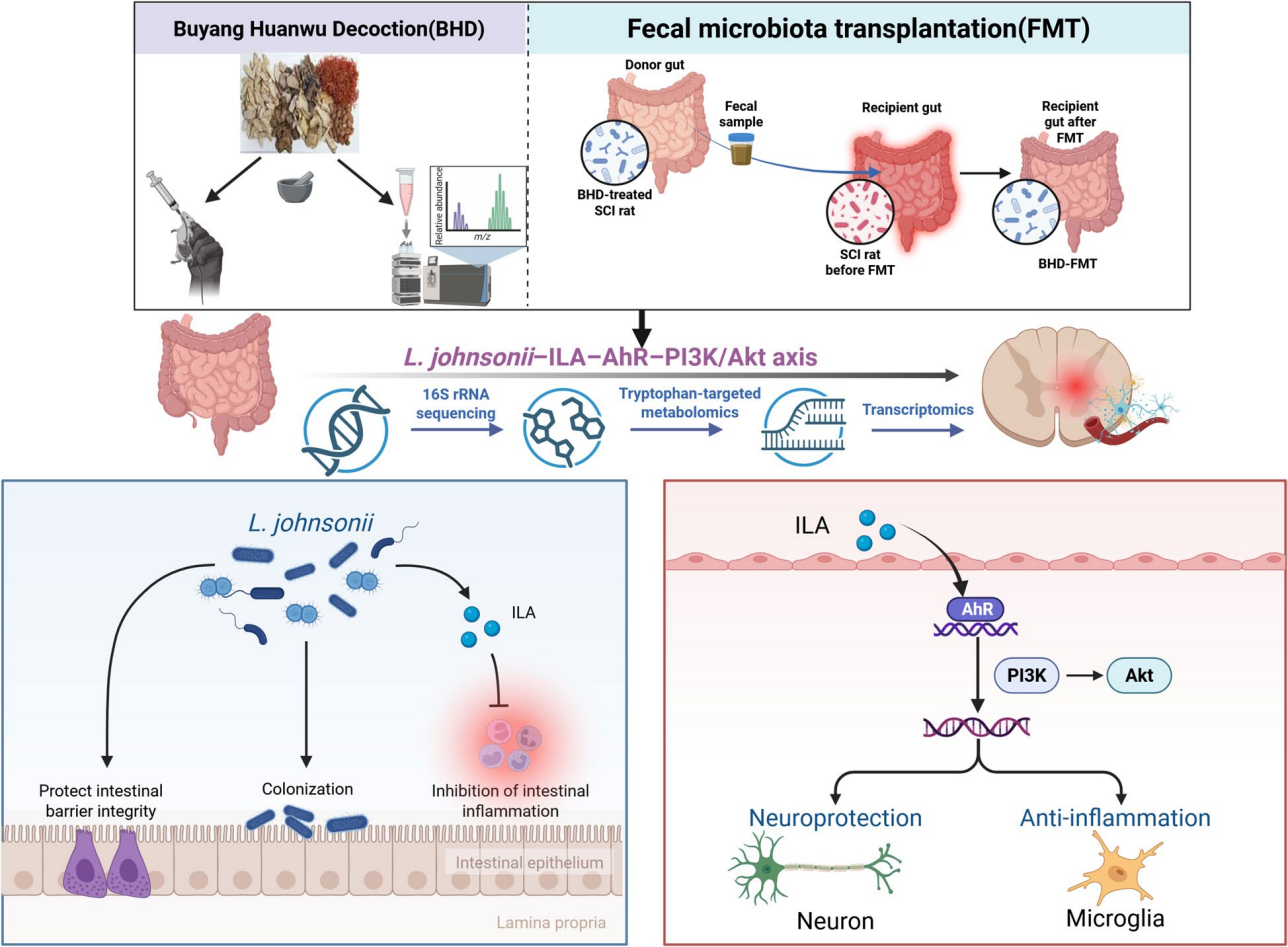

**Supplementary Information:**

The online version contains supplementary material available at 10.1186/s13020-026-01408-x.

## Introduction

Spinal cord injury (SCI) is one of the leading causes of permanent paralysis as well as sexual, bladder, and bowel dysfunction [1]. Globally, approximately 2 to 3 million individuals are affected by SCI, with an additional 250,000 to 500,000 new cases reported annually, imposing a substantial economic burden on patients and society [2]. Currently, the treatment of SCI primarily relies on surgical intervention and rehabilitation training [3], but the therapeutic outcomes remain limited; therefore, novel strategies are urgently needed to promote effective spinal cord repair. In recent years, increasing attention has been paid to the role of the gut microbiota in various neurological disorders [4–6]. Studies have shown that SCI induces gut dysbiosis and disrupts intestinal barrier integrity, while the resulting microbial imbalance further exacerbates spinal inflammation and inhibits tissue repair, thereby forming a bidirectional “gut–spinal cord axis” vicious cycle [7, 8]. Therefore, modulation of the gut microbiota has been proposed as a potential therapeutic target for SCI. Previous studies have demonstrated that the administration of probiotics (VSL#3) effectively restores gut microbial balance and promotes spinal cord repair [9]. However, the clinical translation of probiotic therapy faces challenges, including low survival rate after oral administration, colonization resistance within the dysbiotic gut, and safety concerns regarding optimal strain selection in immunocompromised patients [10–13].

In contrast, Traditional Chinese Medicine (TCM) formulas contain multiple bioactive components that can act synergistically on different microbial populations, increase the relative abundance of probiotics, and reduce the risk of therapeutic failure and metabolic disturbances associated with the low survival rate, colonization difficulty, and lack of consensus regarding probiotic selection in probiotic therapy [14]. Buyang Huanwu Decoction (BHD), a classical formula developed by the renowned Qing Dynasty physician Wang Qingren, is composed of seven herbal medicines, including *Astragalus membranaceus*, *Angelica sinensis*, *Ligusticum chuanxiong*, *Carthamus tinctorius*, *Prunus persica*, *Pheretima aspergillum*, and *Paeonia rubra* [15], and has been shown to effectively treat post-stroke sequelae and neurological dysfunctions [16]. Modern pharmacological studies have revealed that BHD exerts multi-target neuroprotective effects, including the regulation of neuroinflammation, promotion of axonal regeneration, improvement of microcirculation, and modulation of immune responses [17–19]. Previous studies have demonstrated that BHD exerts reparative effects on SCI [20, 21]. Moreover, BHD has been found to ameliorate various central nervous system disorders by modulating the gut microbiota, exhibiting gut microbiota–centered therapeutic potential in models of cerebral ischemia, Alzheimer’s disease, and Parkinson’s disease [22–25]. However, whether BHD can alleviate gut dysbiosis following SCI and exert therapeutic effects through the “gut–spinal cord axis” remains unclear.

BHD is primarily composed of natural plant-derived constituents, and due to the restrictive nature of the blood–brain barrier, most of its active components are unable to directly act on the central nervous system but can enter the gut in large quantities and interact extensively with the intestinal microbiota [25]. The gut microbiota can produce metabolites beneficial for SCI repair, among which the tryptophan metabolic pathway is of particular importance [26, 27]. Previous studies have shown that microbial metabolism of tryptophan produces indole derivatives such as indole-3-lactic acid (ILA) and indole-3-acetic acid (IAA), which serve as natural ligands of the aryl hydrocarbon receptor (AhR) to regulate post-injury inflammatory responses and neuroregeneration [28]. In various neurodegenerative diseases and central inflammatory models, dysregulation of tryptophan metabolism has been recognized as a key metabolic axis linking gut dysbiosis to neurological dysfunction [29]. Therefore, we hypothesize that the neuroprotective effects of BHD may not arise solely from the direct actions of its chemical constituents, but rather from its ability to reshape the gut microbiota composition, enhance tryptophan metabolism, and thereby remotely modulate spinal signaling pathways.

To verify this hypothesis, we first evaluated the effects of direct intragastric administration of BHD on motor function recovery, spinal cord pathology, and intestinal histopathology after SCI, and analyzed its direct impact on the gut microbiota through metagenomic sequencing. Subsequently, fecal microbiota transplantation (FMT) experiments were conducted to exclude the direct pharmacological effects of BHD and to investigate whether its neuroprotective efficacy is mediated by the gut microbiota. Furthermore, an integrative multi-omics approach combining 16S rRNA sequencing, targeted tryptophan metabolomics, and transcriptomics was employed to determine whether BHD exerts therapeutic effects on SCI by reshaping gut microbiota composition, modulating tryptophan-derived metabolites, and regulating key spinal signaling pathways through AhR activation, thereby establishing a specific “gut microbiota–metabolite–signaling pathway” network axis. Finally, the involvement of this network axis in BHD-mediated SCI repair was validated using an AhR inhibitor, aiming to provide a novel perspective for elucidating the microecological mechanisms of traditional Chinese herbal formulas and to explore new therapeutic strategies for the clinical management of SCI.

## Materials and methods

### Animals

A total of 104 adult female Sprague–Dawley rats (6–8 weeks, 250 ± 20 g) were purchased from Chengdu Dossy Experimental Animals Co., Ltd. (animal license number: SCXK (Chuan) 2025-0030) and housed under standard conditions (22 ± 2 °C, 55% ± 10% humidity, 12 h light/dark cycle) with ad libitum access to food and water. All animal procedures were conducted in accordance with the Regulations for the Administration of Affairs Concerning Experimental Animals (Revised by the State Council of the People's Republic of China, 2017) and the Guidelines for the Care and Use of Laboratory Animals issued by the Chinese Association for Laboratory Animal Sciences. The study was approved by the Biomedical Ethics Committee of the Health Science Center of Xi’an Jiaotong University (Approval No. XJTUEA2023-1342).

### Establishment of SCI animal models

Rats were anesthetized with 2% isoflurane (RWD Life Science, Shenzhen, China), and the T10 spinal cord was exposed via laminectomy. A moderate contusion injury (70 kilodynes) was then induced using the Infinite Horizons Impactor (Precision Systems & Instrumentation, Lexington, KY, USA). After the injury, the muscle and skin were carefully sutured. During surgery and recovery, animals were placed in a warming chamber until fully awake. Bladders were manually expressed at least twice daily throughout the study to prevent urinary retention. Given the relative ease of bladder expression and lower risk of urinary tract complications in females compared to males [30], only female rats were included in the present study. All surgical procedures and postoperative care were conducted in accordance with the guidelines for rodent survival surgery established by the Experimental Animal Committee of Xi'an Jiaotong University.

### FMT

Fecal samples were collected and processed as previously described [31]. The donor rats included two groups: (1) SCI rats without treatment (SCI-FMT) and (2) SCI rats treated with BHD (BHD-FMT). Fresh fecal pellets were collected from the donor rats under SPF conditions. Stools from each donor group were pooled separately, and 100 mg of feces was resuspended in 1 mL of sterile saline (100 mg:1 mL). The suspension was vortexed vigorously for 10 s (Vortex-Genie 2, Scientific Industries, USA; speed 9) and then centrifuged at 800×*g* for 3 min. The supernatant was collected and used as transplant material. Donor stool suspensions were freshly prepared on the day of transplantation and administered by oral gavage within 2 h to preserve bacterial viability and composition.

### Experimental groups

Experiment 1: Rats were randomly divided into three groups (n = 8 per group, Fig. 2A): Sham, SCI, and BHD. Sham: Rats underwent T10 laminectomy without spinal cord compression and received vehicle (0.1 mL sterile saline). SCI: Rats received SCI as described above and were given vehicle. BHD: Rats received the same surgical procedure as the SCI group and were treated with BHD (25 g/kg/day, oral gavage) for 21 consecutive days starting on the day of surgery. The BHD dose was selected based on previous studies demonstrating its efficacy and safety in SCI models [20]. This dose was employed for the present study, which aimed to investigate the novel gut–spinal cord axis mechanism rather than establish a dose–response relationship. It provided a robust therapeutic window for our multi-omics and FMT experiments while ensuring animal welfare and minimizing potential confounders associated with high-volume gavage over the extended treatment period. Throughout the study, rats were manually voided three times daily, and the perineum was regularly cleaned and kept dry.

Experiment 2: Rats were randomly assigned to four groups (n = 10 per group, Fig. 5A): Sham, SCI-FMT, BHD-FMT, and BHD. Sham: Rats underwent T10 laminectomy without spinal cord compression and received vehicle (0.1 mL sterile saline). SCI-FMT: Rats underwent SCI and subsequently received FMT from SCI donor rats for 21 consecutive days. BHD-FMT: Rats underwent SCI and subsequently received FMT from SCI + BHD donor rats for 21 consecutive days. BHD: Rats underwent SCI and were treated directly with BHD (25 g/kg/day, oral gavage) for 21 consecutive days. Prior to FMT, recipient rats in the SCI-FMT and BHD-FMT groups were pretreated with a broad-spectrum antibiotic cocktail via drinking water for 2 weeks to deplete resident gut microbiota. The cocktail consisted of vancomycin (0.1 g/L; Sigma-Aldrich, USA), neomycin (0.2 g/L; Macklin, China), metronidazole (0.2 g/L; Solarbio, China), and ampicillin (0.2 g/L; Solarbio, China). After antibiotic treatment, 100 μL of freshly prepared fecal suspension (donor stool resuspended in sterile saline) or vehicle was administered daily by oral gavage for 21 consecutive days.

Experiment 3: Rats were randomly assigned to four groups (n = 10 per group, Fig. 10A): Sham, SCI-FMT, BHD-FMT, and BHD-FMT + AhRi. The first three groups were established as described in Experiment 2. BHD-FMT + AhRi: Rats received the same treatment as the BHD-FMT group, with the addition of the AhR inhibitor CH-223191 (10 mg/kg, intraperitoneal injection, once daily; Sigma-Aldrich, USA) administered concurrently for 21 consecutive days.

### Preparation of BHD

BHD was prepared according to traditional decoction procedures. First, 60 g of *Angelica sinensis* (Lot: 241201) and 30 g of *Rhizoma Chuanxiong* (Lot: 240302-2) were boiled six times in 540 mL of water, and 200 mL of distillate was collected and filtered for further use. Subsequently, 1200 g of *Astragalus membranaceus* (Lot: 225122901), 60 g of *Radix Paeoniae Rubra* (Lot: 240601-1), 30 g of *Pheretima aspergillum* (Lot: 240902-1), 30 g of *Carthamus tinctorius* (Lot: 241101-1), and 30 g of *Prunus persica* (Lot: 250701-1) were mixed with 10,800 mL of water and decocted twice for 1 h each. The resulting extracts were filtered and combined, then concentrated under reduced pressure using a rotary evaporator to a volume of approximately 400 mL. This concentrate was mixed with 200 mL of the previously collected distillate, 3 mL of Tween-80, and distilled water to a final volume of 600 mL. The final extract was a dark brown solution, in which 1 mL corresponded to 2.4 g of crude raw material. The preparation was sterilized by high-pressure steam and stored in sterile 250-mL sealed infusion bottles at 4 °C. All dried crude herbs were purchased from the Zhang Zhongjing Pharmaceutical Company in Henan Province, China. All decoction and formulation procedures were performed at Honghui Hospital, Xi’an Jiaotong University, Xi’an, China.

### Liquid chromatography–mass spectrometry (LC–MS/MS) analysis

Metabolite Extraction of TCM Solutions: The TCM solutions were centrifuged at 12,000 rpm (RCF = 13,800 × g, R = 8.6 cm) for 15 min at 4 °C. Three hundred microliters of the supernatant were mixed with 1000 μL of extraction solution (MeOH:ACN:H₂O, 2:2:1, v/v/v) containing deuterated internal standards. The mixture was vortexed for 30 s, sonicated for 5 min in a 4 °C water bath, and incubated at − 20 °C for 1 h to precipitate proteins. The samples were then centrifuged at 12,000 rpm (RCF = 13,800 × g, R = 8.6 cm) for 15 min at 4 °C. The resulting supernatants were filtered through a 0.22 μm membrane and transferred to fresh glass vials for analysis. Quality control (QC) samples were prepared by mixing equal aliquots of the supernatants from all samples.

LC–MS/MS Analysis: LC–MS/MS analyses were performed using an UHPLC system (Vanquish, Thermo Fisher Scientific) coupled to a Phenomenex Kinetex C18 column (2.1 mm × 100 mm, 2.6 μm) and an Orbitrap Exploris 120 mass spectrometer (Thermo Fisher Scientific). Mobile phase A consisted of 0.01% acetic acid in water, and mobile phase B consisted of IPA:ACN (1:1, v/v). The autosampler temperature was maintained at 4 °C, and the injection volume was 2 μL. The Orbitrap Exploris 120 was operated in data-dependent acquisition (DDA) mode under control of Xcalibur software (Thermo Fisher Scientific). The ESI source parameters were set as follows: sheath gas flow rate, 50 Arb; auxiliary gas flow rate, 15 Arb; capillary temperature, 320 °C; full MS resolution, 60,000; MS/MS resolution, 15,000; normalized collision energy (NCE), 20/30/40; spray voltage, 3.8 kV (positive) or − 3.4 kV (negative).

Data Preprocessing and Annotation: The raw data were converted to mzXML format using ProteoWizard and processed with an in-house program. The R package and PSNGM were used for metabolite identification.

### Hematoxylin‒eosin (H&E) staining

Histological analysis was performed via H&E staining. At 21 days postinjury, the rats were deeply anesthetized with 3% pentobarbital sodium (Solarbio, Beijing, China) and transcardially perfused with saline followed by 4% paraformaldehyde (Solarbio, Beijing, China). A 10-mm spinal cord segment encompassing the lesion epicenter (5 mm rostral and 5 mm caudal) and colonic tissues were carefully harvested and fixed in 4% paraformaldehyde (Solarbio, Beijing, China) at 4 °C overnight. The tissues were then embedded in paraffin and sectioned at a thickness of 5 μm via a microtome (Leica, Wetzlar, Germany). The sections were deparaffinized in xylene, rehydrated through a graded ethanol series, and stained with hematoxylin (Solarbio, Beijing, China) for 5 min. After being rinsed in distilled water and differentiated, the sections were counterstained with eosin (Solarbio, Beijing, China) for 5 min, dehydrated with increasing ethanol concentrations (85% and 95%), cleared in xylene, and mounted with neutral gum. Histological images were captured via a BX53F2 light microscope (Olympus, Tokyo, Japan).

### Behavioral tests

The motor function of the rats was observed at 1 day, 2 days, 3 days, 4 days, 1 week, 2 weeks, and 3 weeks after SCI. All observations and data analyses were conducted in a double-blind manner, where the evaluators were unaware of the rat's identification number and group assignment. Two researchers performed the functional assessments, and the final score for each rat was determined by consensus between the two researchers. In the case of a disagreement, a third independent evaluator was invited to participate in the assessment. The same researchers were responsible for the entire process of functional assessment, and no personnel changes or substitutions were allowed midway.

Motor function was evaluated using the Basso, Beattie, Bresnahan (BBB) locomotor rating scale. The BBB scale is a 21-point scale (0–21) specifically designed and validated for assessing open-field locomotion in rats with spinal cord injury. It evaluates hindlimb joint movement, weight support, stepping, coordination, and trunk stability. A score of 0 indicates no observable hindlimb movement, while a score of 21 represents normal locomotion. Assessments were conducted at the specified time points (1, 2, 3, 4, 7, 14, and 21 days post-injury), and the final BBB score for each rat was recorded.

The inclined plane test was performed to assess limb strength and postural stability. The apparatus consisted of an adjustable inclined plane (60 cm × 30 cm) covered with a non-slip rubber mat. For rats with severe spinal cord injury (SCI), a baseline deficit was established by setting the initial testing angle to 20° on the first day post-injury, as pilot studies confirmed that this was the maximum angle they could maintain. At each test time point, rats were gently placed on the plane, with their body axis parallel to the incline and their head facing upwards. The angle was then increased in increments of 5°. At each angle, the rat was observed for up to 5 s. The maximum angle at which the rat could maintain its position without falling or slipping for a full 5 s was recorded as the score for that trial. Each rat underwent five consecutive trials at each time point, with a 1-min rest between trials, and the mean of these five scores was calculated as the final value for statistical analysis.

### Quantitative PCR (qPCR)

In accordance with the manufacturer's manual, total RNA was extracted from the spinal cord using the TRIzol reagent (Invitrogen Life Technologies, Carlsbad, USA). Quantitative analysis was accomplished via a two-step reaction procedure: reverse transcription (RT) and PCR. Each RT reaction encompassed 0.5 μg RNA, 2 μL of 5 × TransScript All-in- one SuperMix for qPCR, and 0.5 μL of gDNA remover, with a total volume of 10 μL. The reaction was conducted on the PCR system 9700 (Applied Biological Systems, USA) at 42 °C for 15 min, followed by 5 s at 85 °C. Subsequently, the 10 μL RT reaction mixture was diluted tenfold in nuclease-free water and incubated at − 20 °C. Real-time PCR was carried out using the LightCycler 480 II real-time PCR system (Roche, Switzerland), where 10 μL of the PCR mixture comprised 1 μL of cDNA, 5 μL of 2 × PerfectStartTM green qPCR SuperMix (TransGen Biotech, Beijing, China), 0.2 μL of the forward primer, 0.2 μL of the reverse primer, and 3.6 μL of nuclease-free water. The reaction process was incubated at 94 °C for 0.5 min, followed by 5 s at 94 °C and 30 s at 60 °C for 45 cycles. All specimens were analyzed thrice. After the PCR cycle, melting curve analysis was performed to verify the specific generation of the anticipated PCR product. The primer sequences are listed in Table S1. Finally, the relative mRNA expression level of each target gene was normalized to the endogenous reference gene *Gapdh* and calculated using the 2^−ΔΔCt^ method relative to the sham-operated control group.

### Immunofluorescence staining

The spinal cord and colonic tissues were fixed with 4% paraformaldehyde (Solarbio, Beijing, China), paraffin-embedded, and sectioned. The slices were dewaxed and rehydrated. After antigen retrieval, sections were blocked with 5% BSA in PBS for 1 h at room temperature. Subsequently, sections were incubated overnight at 4 °C with the following primary antibodies diluted in blocking buffer: rabbit polyclonal anti-NeuN (1:100, 26975-1-AP; Proteintech, Wuhan, China), rabbit polyclonal anti-synapsin (1:100, 17785-1-AP; Proteintech), rabbit polyclonal anti-NF-200 (1:100, 18934-1-AP; Proteintech), rabbit polyclonal anti-ZO-1 (1:100, 21773-1-AP; Proteintech), or rabbit monoclonal anti-occludin (1:100, 27260-1-AP; Proteintech). After washing with PBS, sections were incubated with appropriate fluorophore-conjugated secondary antibodies for 1 h at room temperature in the dark. Nuclei were counterstained with DAPI. After final washes, slides were mounted with anti-fade mounting medium and examined using a fluorescence microscope. The relative fluorescence intensity was quantified using Image-Pro Plus 7.0 (Media Cybernetics, Silver Spring, MD, USA).

### Gastrointestinal transit assessment

The rats were fasted for 24 h and then refed with 10 g of standard chow for 30 min. Following refeeding, 0.2 mL of 1% edible dye (Brilliant Blue FCF, Aladdin Biochemical Technology Co., Ltd., Shanghai, China) was administered via oral gavage. At 2, 3, and 4 h post gavage, animals were euthanized by intraperitoneal injection of an overdose of pentobarbital sodium (150 mg/kg), and the entire gastrointestinal tract was carefully excised. In Experiment 1 (8 rats/group), sampling was performed at 2 h (n = 2), 3 h (n = 3), and 4 h (n = 3); in Experiment 2 (10 rats/group), at 2 h (n = 3), 3 h (n = 3), and 4 h (n = 4). The distribution of the orally administered dye within the small intestine was recorded. Gastrointestinal motility was assessed using two quantitative parameters: (1) the distance from the pylorus to the trailing edge of the dye, which reflected the progression of intestinal contents, and (2) the intestinal transit rate (%), calculated as follows: transit rate (%) = distance from pylorus to the leading edge of the pigment/total length of the small intestine × 100%.

### Metagenomic sequencing and analysis

Fecal samples from rats in the Sham, SCI, and BHD groups of Experiment 1 were subjected to metagenomic analysis to investigate gut microbial composition and structure. Total microbial genomic DNA was extracted using the OMEGA Soil DNA Kit (D5625-01) according to the manufacturer’s protocol, and DNA quality was assessed with a NanoDrop ND-1000 spectrophotometer (Thermo Fisher Scientific, USA) and agarose gel electrophoresis. Sequencing libraries (~ 400 bp insert size) were prepared with the Illumina TruSeq Nano DNA LT Library Preparation Kit and sequenced on the Illumina HiSeq X-ten platform (PE150; Illumina, USA) by Personal Biotechnology Co., Ltd. (Shanghai, China). Raw reads were quality-filtered using Cutadapt (v1.2.1) [32] and fastp (v0.20.0) [33], and host-derived reads were removed by alignment to the Rattus norvegicus reference genome (Rnor_6.0) using BMTagger. Clean reads were taxonomically classified with Kraken2 (v2.1.2) [34] against the RefSeq database (release July 2024). De novo assembly was performed using MEGAHIT (v1.1.2) [35], and open reading frames were predicted by MetaGeneMark (v3.38) [36]. A non-redundant gene catalog was constructed using mmseqs2 (v12-113e3) [37], and gene abundance was quantified by mapping reads to this catalog with Salmon (v1.5.0), normalized as CPM. Functional annotation was performed against KEGG, EggNOG, and CAZy databases using mmseqs2 and EggNOG-mapper (v2) [38].

### 16S rRNA gene sequencing and analysis

Fecal samples from rats in the Sham, SCI-FMT and BHD-FMT groups of Experiment 2 were subjected to 16S rRNA sequencing to characterize gut microbial communities. Total genomic DNA was extracted using the MagBeads FastDNA Kit for Soil (MP Biomedicals, CA, USA) following the manufacturer’s protocol, and DNA integrity was assessed by agarose gel electrophoresis and quantified with a NanoDrop NC2000 spectrophotometer (Thermo Fisher Scientific, USA). The V3–V4 region of the bacterial 16S rRNA gene was amplified with primers 338 F (5′-ACTCCTACGGGAGGCAGCA-3′) and 806R (5′-GGACTACHVGGGTWTCTAAT-3′). PCR products were purified using VAHTS DNA Clean Beads (Vazyme, China), quantified with the Quant-iT PicoGreen dsDNA Assay Kit (Invitrogen, USA), pooled in equimolar concentrations, and sequenced on the Illumina NovaSeq 6000 platform (paired-end 2 × 250 bp; Illumina, USA) by Personal Biotechnology Co., Ltd. (Shanghai, China).

Raw sequencing reads were processed with QIIME2 (v2024.5) [39]. Briefly, sequences were demultiplexed, adapters removed using Cutadapt (Martin, 2011), and denoised, merged, and chimera-checked using the DADA2 plugin (Callahan et al., 2016) to obtain amplicon sequence variants (ASVs). ASVs were aligned with MAFFT [40] and used to construct a phylogenetic tree with FastTree2 [41]. Alpha diversity indices (Chao1, Shannon, Simpson, Faith’s PD, Pielou’s evenness, and Good’s coverage) and beta diversity metrics (Jaccard, Bray–Curtis, weighted UniFrac, and unweighted UniFrac) were calculated after rarefaction and visualized with QIIME2 and R (v4.3.3). Taxonomic assignment of ASVs was performed with the SILVA database (release 138) using the naïve Bayes classifier in QIIME2 [42]. Group differences in microbial community composition were assessed using PERMANOVA (Anderson, 2001) based on beta diversity distance matrices. Differentially abundant taxa were identified with LEfSe [43], and random forest analysis was used to classify groups based on genus-level abundance profiles. Functional predictions were inferred using PICRUSt2 against KEGG and MetaCyc databases.

### Targeted LC–MS/MS analysis of tryptophan metabolites

Fecal metabolites related to tryptophan metabolism were profiled in the four groups of rats from Experiment 2 using targeted LC–MS/MS analysis. Briefly, 50 mg of fecal sample was homogenized in 500 μL of methanol containing 20 μL of internal standard solution (250 ng/mL). The mixture was vortexed for 3 min, incubated at − 20 °C for 30 min, and centrifuged at 12,000 rpm for 10 min at 4 °C. The supernatant was collected, centrifuged again under the same conditions, and 150 μL of the clarified extract was used for analysis. Chromatographic separation was carried out on a Waters ACQUITY UPLC HSS T3 C18 column (100 × 2.1 mm, 1.8 μm) with a mobile phase of 0.1% formic acid in water (A) and acetonitrile with 0.1% formic acid (B), using a gradient elution (10% B for 0–1 min, 10–95% B for 1–6 min, 95% B for 6–7 min, and re-equilibration at 10% B for 7.1–10 min). The flow rate was 0.35 mL/min, column temperature 40 °C, and injection volume 2 μL. Mass spectrometric detection was performed on a QTRAP® 6500 + LC–MS/MS system (Sciex, USA) equipped with an electrospray ionization (ESI) source operating in both positive and negative ion modes. Source parameters were as follows: temperature, 550 °C; ion spray voltage, + 5500 V/− 4500 V; and curtain gas, 35 psi. Data were acquired in scheduled multiple reaction monitoring (MRM) mode for tryptophan and its metabolites using Analyst 1.6.3 software (Sciex). Quantification was performed with MultiQuant 3.0.3 (Sciex), and mass spectrometer parameters (declustering potential and collision energy) were optimized for each metabolite.

### RNA-Seq analysis

For transcriptome profiling, spinal cord tissues were collected from rats in the Sham, SCI-FMT, and BHD-FMT groups of Experiment 2, as well as from the Sham, SCI-FMT, BHD-FMT, and BHD-FMT + AhRi groups of Experiment 3. Total RNA was extracted using TRIzol Reagent (Invitrogen Life Technologies, Carlsbad, USA), and RNA concentration, quality, and integrity were assessed with a NanoDrop spectrophotometer (Thermo Fisher Scientific, Waltham, USA). Three micrograms of total RNA from each sample were used to construct sequencing libraries. Briefly, mRNA was purified from total RNA using poly-T oligo-attached magnetic beads and fragmented with divalent cations at elevated temperature. First-strand cDNA was synthesized with random oligonucleotides and SuperScript II, followed by second-strand cDNA synthesis using DNA polymerase I and RNase H. After end-repair and adenylation of 3′ ends, Illumina PE adapters were ligated. Libraries of 400–500 bp fragments were selected and purified using the AMPure XP system (Beckman Coulter, USA). Enriched DNA fragments were amplified with Illumina PCR Primer Cocktail (15 cycles), purified again with AMPure XP beads, and quantified with an Agilent Bioanalyzer 2100 (Agilent Technologies, USA). Finally, sequencing was performed on the Illumina NovaSeq 6000 platform (Illumina, San Diego, USA) at Shanghai Personal Biotechnology Co., Ltd.

### Western blot analysis

For Western blotting, the same spinal cord samples used for transcriptome analysis were examined (Experiment 2: Sham, SCI-FMT, and BHD-FMT groups; Experiment 3: Sham, SCI-FMT, BHD-FMT, and BHD-FMT + AhRi groups). A 1-cm segment of spinal cord (T9–T11, with the injury epicenter at the middle and extending 5 mm rostrally and caudally) was collected. Total protein was extracted by homogenizing the tissue in lysis buffer for 1 h, followed by centrifugation at 14,000×*g* for 8 min at 4 °C. Protein concentrations in the supernatant were determined using a BCA protein assay kit (Thermo, 23227, China). Equal amounts of protein (50 μg) were separated by 12% SDS–polyacrylamide gel electrophoresis and transferred onto PVDF membranes. After blocking with 5% nonfat milk in TBST for 1 h, the membranes were incubated overnight at 4 °C with primary antibodies against AHR (1:1000, MA1-513, Thermo), PI3K (1:2000, 60225-1-Ig, Proteintech), Akt (1:2000, 10176, Proteintech), and P65 (1:4000, 80979, Proteintech). Following three washes in TBST, membranes were incubated with appropriate HRP-conjugated secondary antibodies. GAPDH (1:200000, 60004, Proteintech) served as a loading control. Protein bands were visualized using an enhanced chemiluminescence system and imaged with a ChemiDoc MP System (Bio-Rad, Hercules, CA, USA). Band intensities were quantified with Quantity One software (Bio-Rad, Hercules, CA, USA).

### Multiomics analysis and network construction

To explore the potential mechanisms underlying spinal cord repair, a multiomics integration analysis was performed using data from Experiment 2 (SCI-FMT and BHD-FMT groups). Differential gut microbial species, metabolites, and spinal cord genes between the two groups were identified and used to construct a gut–spinal cord regulatory network. Spearman correlation coefficients were calculated between microbial species, metabolites, and differentially expressed genes, with significant correlations defined as |r|> 0.8 and p < 0.05. In the resulting network, nodes represented microbial species, metabolites, or genes, while edges represented significant correlations. The network was visualized and analyzed using Cytoscape (v3.10.2), with node size and color indicating connectivity and type, respectively. Key hubs and interactions were further examined to reveal potential regulatory pathways of the gut–spinal cord axis.

### Statistical analyses

All data are presented as means ± SD. Two-group comparisons were performed using unpaired Student’s t-test for normally distributed data or Mann–Whitney U test for non-normally distributed data. Categorical variables were compared using the chi-square test or Fisher’s exact test when expected counts were < 5. Multiple group comparisons were performed using one-way ANOVA with Tukey’s post-hoc test. Repeated measures data were analyzed by two-way ANOVA with Bonferroni correction. Differential analyses of metagenomic and metabolomic data were conducted using the Limma package in R on normalized abundance matrices, with P-values adjusted for multiple testing using the Benjamini–Hochberg method. All statistical analyses were performed using GraphPad Prism 7.0 (GraphPad Software, La Jolla, CA, USA) or R software (version 4.3.3). Differences were considered significant at an adjusted P < 0.05.

## Results

### LC‒MS/MS analysis of BHD components

**Fig. 1:**
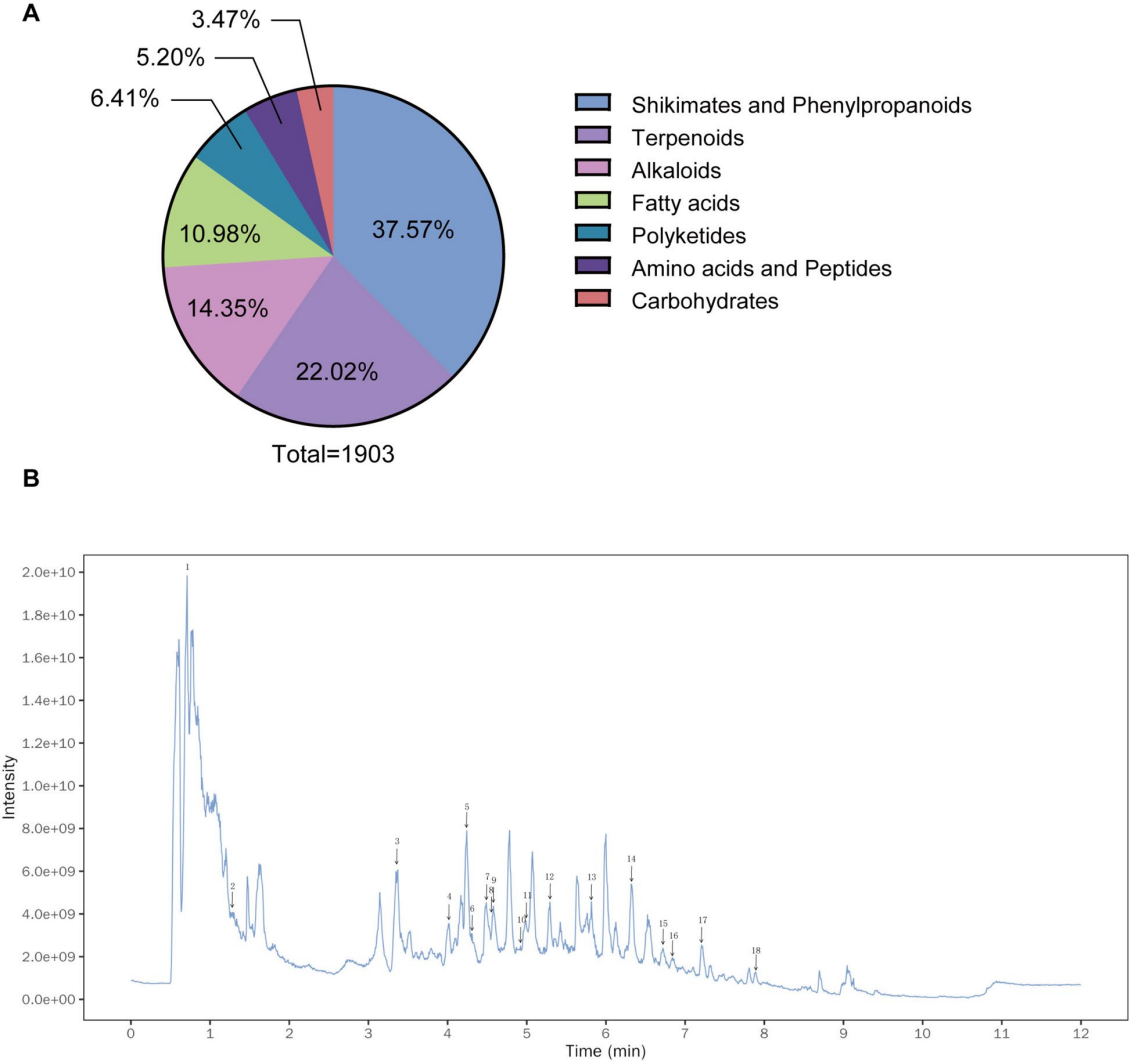
Fingerprint analysis of BHD by LC–MS/MS. **A** Superclass distribution of all metabolites identified in BHD by LC–MS/MS. **B** Total ion chromatogram (TIC) of BHD in positive ion mode, with representative peaks annotated for classical components. 1. Trigonelline; 2. Crotonoside; 3. Tryptophan; 4. Amygdalin; 5. Albiflorin; 6. Hydroxysafflor yellow A; 7.Baimaside; 8. Paeoniflorin; 9. Glycitin; 10. 4-Hydroxycinnamic acid; 11. Ferulate; 12. Ononin; 13. Gnaphaliin; 14. Isoformononetin; 15. Astragaloside IV; 16. Astragaloside II; 17. Angeolide; 18. Soyasaponin I

To comprehensively characterize the chemical composition of BHD, LC–MS/MS analysis was performed in both positive and negative ion modes. A total of 1903 compounds were detected, encompassing various chemical superclasses. The overall superclass distribution of all identified metabolites is illustrated in Fig. 1A, indicating that shikimic acid and phenylpropanoid derivatives, terpenoids, alkaloids, fatty acids, polyketides, amino acids and short peptides, and carbohydrates constitute the major classes. Notably, the positive ion mode resulted in a greater response intensity and broader coverage for the classical constituents of BHD identified in this study (Fig. 1B), including 18 active compounds such as trigonelline, paeoniflorin, hydroxysafflor yellow A, albiflorin, mangiferin, and astragaloside IV. Table 1 provides detailed fingerprint information for these compounds, including retention times, exact m/z values, molecular formulas, and structural classifications.

**Table 1:** Representative compounds identified from BHD by LC–MS/MS analysis

MS2_name	Formula	Superclass	Subclass	mzmed	rtmed	ms2Adduct
Trigonelline	C7H7NO2	Alkaloids	Pyridine alkaloids	138.0551	0.72	[M + H] +
Crotonoside	C10H13N5O5	Carbohydrates	Purine nucleos(t)ides	284.0994	1.33	[M + H] +
Tryptophan	C11H12N2O2	Amino acids and Peptides	Aminoacids	205.0975	3.36	[M + H] +
Amygdalin	C20H27NO11	Amino acids and Peptides	Cyanogenic glycosides	458.1666	4.00	[M + H] +
Albiflorin	C23H28O11	Terpenoids	Pinane monoterpenoids	481.1703	4.24	[M + H] +
Hydroxysafflor yellow A	C27H32O16	Shikimates and Phenylpropanoids	Chalcones	613.1769	4.28	[M + H] +
Baimaside	C27H30O17	Shikimates and Phenylpropanoids	Flavonols	627.1568	4.45	[M + H] +
Paeoniflorin	C23H28O11	Terpenoids	Pinane monoterpenoids	481.1711	4.51	[M + H] +
Glycitin	C22H22O10	Shikimates and Phenylpropanoids	Isoflavones	447.1289	4.59	[M + H] +
4-Hydroxycinnamic acid	C9H8O3	Shikimates and Phenylpropanoids	Cinnamic acids and derivatives	147.0441	4.98	[M-H2O + H] +
Ferulate	C10H10O4	Shikimates and Phenylpropanoids	Cinnamic acids and derivatives	195.0655	4.89	[M + H] +
Ononin	C22H22O9	Shikimates and Phenylpropanoids	Isoflavones	431.1338	5.29	[M + H] +
Gnaphaliin	C17H14O6	Shikimates and Phenylpropanoids	Flavonols	315.0865	5.82	[M + H] +
Isoformononetin	C16H12O4	Shikimates and Phenylpropanoids	Isoflavones	269.0807	6.32	[M + H] +
Astragaloside IV	C41H68O14	Terpenoids	Cucurbitane triterpenoids	785.4686	6.71	[M + H] +
Astragaloside II	C43H70O15	Terpenoids	Cucurbitane triterpenoids	827.4792	6.88	[M + H] +
Angeolide	C24H28O4	Polyketides	Phthalide derivatives	381.2062	7.19	[M + H] +
Soyasaponin I	C48H78O18	Terpenoids	Oleanane triterpenoids	943.5267	7.90	[M + H] +

### BHD improves motor function recovery after SCI by suppressing inflammation and promoting neuroregeneration

**Fig. 2:**
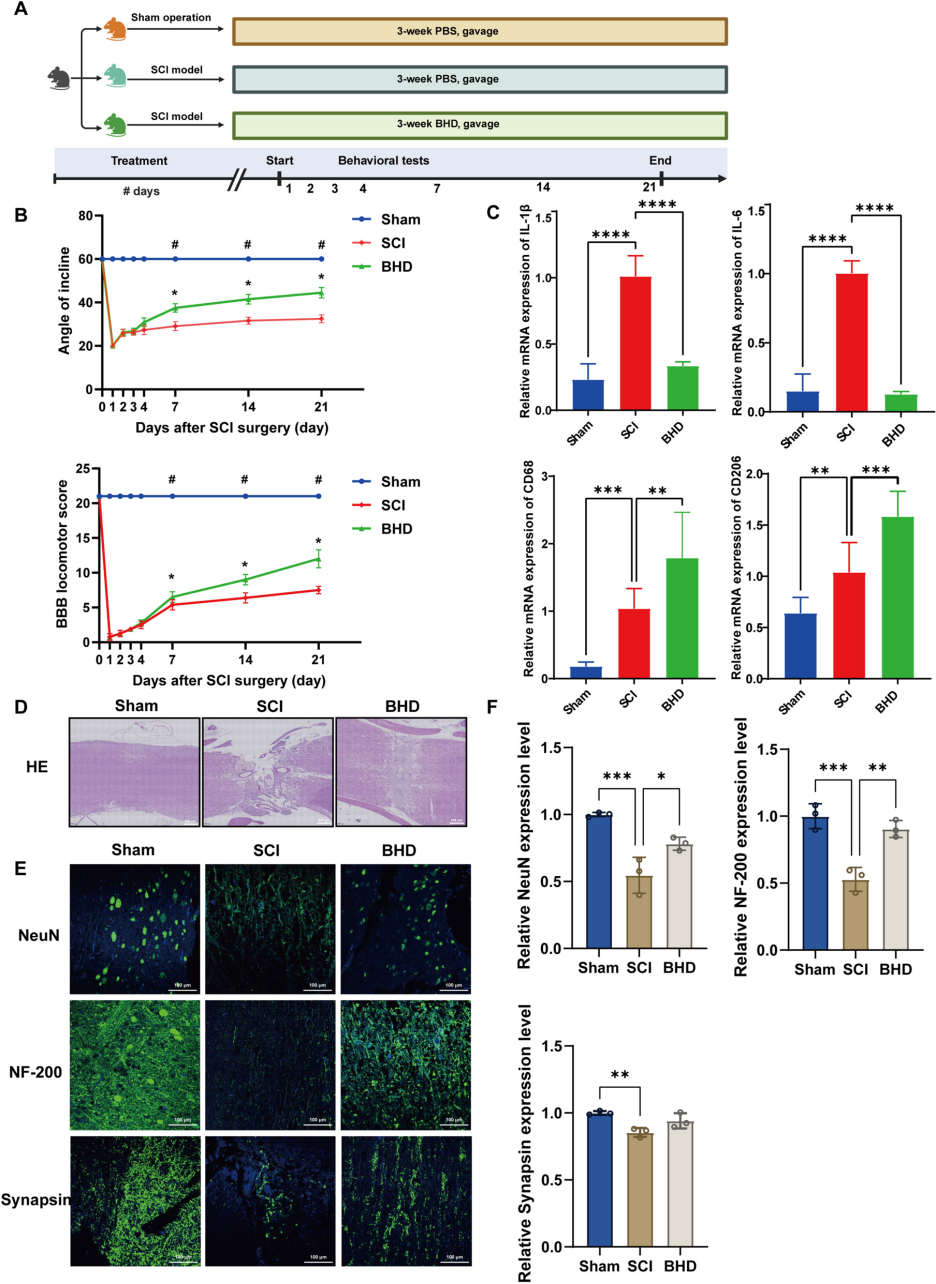
BHD improves motor function recovery after SCI and promotes inflammation suppression and neuroregeneration. **A** Schematic diagram of the experimental workflow and group design. **B** Motor function assessed by inclined plane test and BBB scoring at indicated days post-injury (n = 8). *, Sham vs. SCI; #, BHD vs. SCI (*p* < 0.05). Behavioral scores were analyzed by two-way repeated measures ANOVA followed by Sidak’s post hoc test. **C** qPCR analysis of mRNA expression levels of inflammatory cytokines (*IL-1β* and *IL-6*) and macrophage-associated markers (*CD68* and *CD206*) in spinal cord tissues (n = 3). **D** Representative H&E stained spinal cord sections showing histopathological changes in each group 21 days after injury (n = 3). **E** Immunofluorescence staining of spinal cord tissues for NeuN, NF-200, and Synapsin (green), with DAPI (blue) used for nuclear counterstaining. NeuN staining indicates neuronal survival in the ventral horn, while NF-200 and Synapsin staining reflect axonal regeneration and synaptic preservation (n = 3). **F** Quantitative analysis of NeuN-positive cells and expression levels of Synapsin and NF-200 among the three groups (n = 3). Data are presented as mean ± SD. Differences among multiple groups were analyzed by one-way ANOVA followed by Tukey’s post hoc test. ns: no significance; *p < 0.05, **p < 0.01, ***p < 0.001, ****p < 0.0001

Given the complex composition and multi-target pharmacological properties of BHD, its therapeutic effects on SCI were first evaluated using a direct intragastric administration protocol (Fig. 2A). Inclined plane tests and BBB scoring were performed on days 1, 2, 3, 4, 7, 14, and 21 post-injury (Fig. 2B). The results demonstrated that, starting from day 4 of treatment, rats in the BHD group exhibited significantly improved motor function compared with the SCI control group (*p* < 0.05), and this improvement persisted until day 21. Histological analysis further confirmed these functional improvements. H&E staining (Fig. 2D) revealed that the spinal cord structure remained intact in the sham group, whereas the SCI group exhibited severe tissue disruption and massive inflammatory cell infiltration. In contrast, the BHD-treated group showed reduced inflammation, partial restoration of tissue architecture, and a smaller lesion area (Supplementary Fig.  1A). Regarding the inflammatory response, qPCR analysis (Fig. 2C) revealed that the pro-inflammatory cytokines *IL-1β* and *IL-6* were markedly upregulated in the SCI group but were significantly downregulated following BHD treatment (*p* < 0.0001). Meanwhile, the expression of macrophage-associated genes was altered. BHD intervention increased the expression of the macrophage marker *CD68*, indicating enhanced immune cell infiltration at the injury site. Concurrently, BHD treatment significantly upregulated the expression of *CD206*, a gene associated with anti-inflammatory functions.

With respect to neural repair, immunofluorescence staining (Fig. 2E and F) showed normal ventral horn neurons in the sham group, characterized by large somata and intact axons. Following SCI, the number of NeuN-positive neurons was markedly reduced. However, compared with the SCI group, the number of NeuN-positive neurons was significantly increased in the BHD-treated group (*p* < 0.05). Neurofilament-200 (NF-200) is a cell type-specific protein abundantly present in neuronal axons, and its staining has been widely used to evaluate neuronal and axonal injury [44, 45]. Immunofluorescence staining for NF-200 demonstrated a significant increase in NF-200-positive fibers in the white matter adjacent to the lesion site in the BHD-treated group compared with the SCI group (*p* < 0.01). Synapsin (SYN) is a vesicle protein that serves as a marker of presynaptic membranes [46]. Three weeks after SCI, SYN expression was reduced in the SCI group but markedly enhanced following BHD treatment. Collectively, these findings indicate that BHD markedly improves motor function, suppresses inflammatory responses, and promotes neuronal survival, synaptic preservation, and axonal regeneration in rats with SCI, highlighting its therapeutic potential in mitigating secondary spinal cord injury and providing a foundation for further mechanistic investigations.

### BHD ameliorates gastrointestinal dysfunction, suppresses intestinal inflammation, and enhances barrier function following SCI

**Fig. 3:**
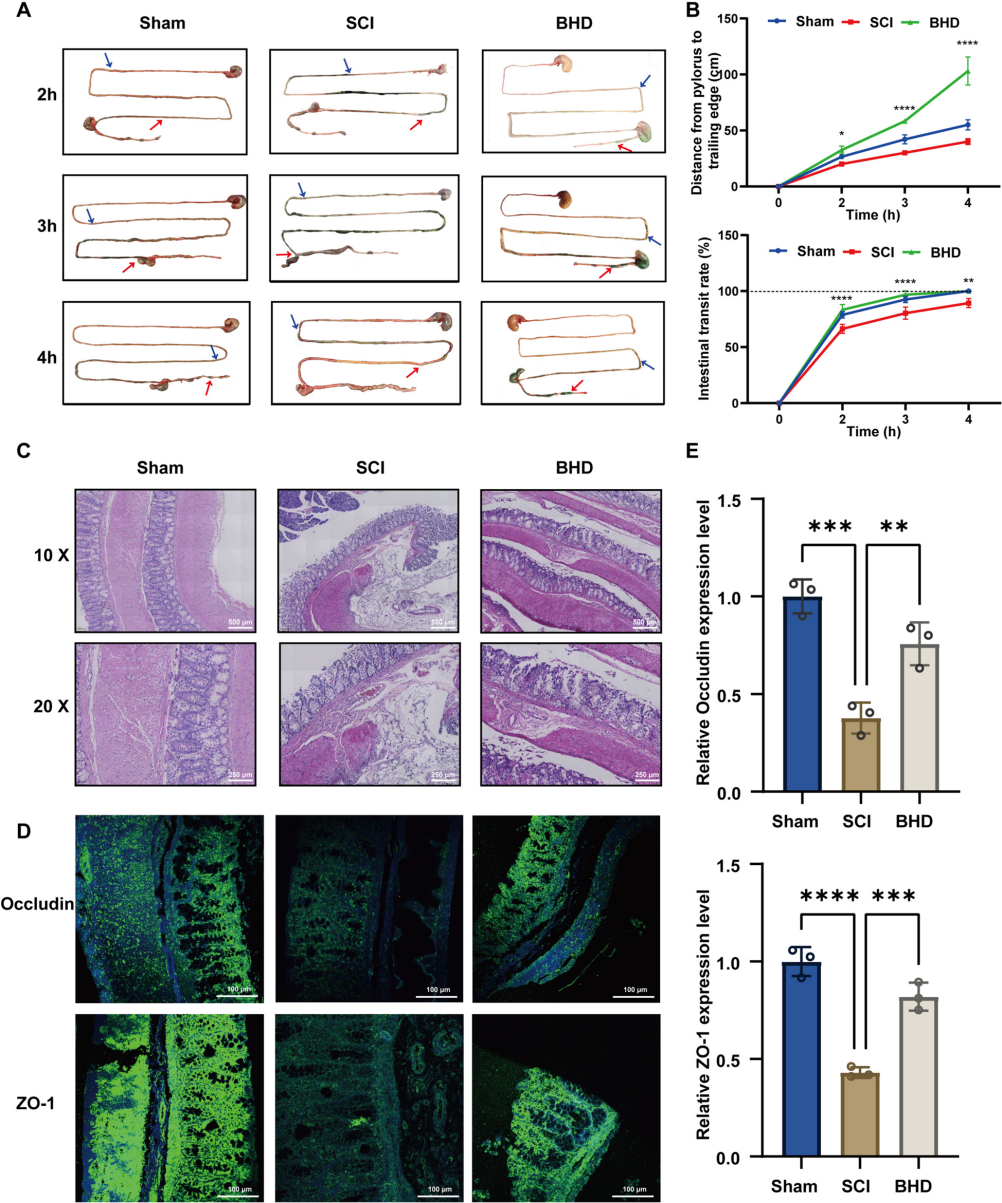
BHD ameliorates gastrointestinal motility disorders and restores intestinal barrier function following SCI. **A** Representative images of gastrointestinal dye distribution at indicated time points post-gavage (2 h, 3 h, and 4 h). Blue and red arrows indicate the proximal and distal ends of the intestinal dye front, respectively. **B** Quantitative analysis of gastrointestinal motility in rats at 2, 3, and 4 h after oral administration of edible dye. Parameters included the distance from the pylorus to the trailing edge of the dye and the intestinal transit rate (%). **C** H&E staining of the colon showing colonic tissue structure and inflammatory infiltration in different groups of rats (n = 3). **D** Immunofluorescence staining of ZO-1 and occludin reflecting changes in tight junction protein expression and localization (n = 3). **E** Quantitative analysis of occludin and ZO-1 expression (n = 3). Data are presented as mean ± SD. Differences among multiple groups were analyzed by one-way ANOVA followed by Tukey’s post hoc test. ns: no significance; *p < 0.05, **p < 0.01, ***p < 0.001, ****p < 0.0001

SCI frequently induces gastrointestinal motility disorders and delayed transit, accompanied by inflammatory responses and disruption of the intestinal barrier [47]. To evaluate the effect of BHD, gastrointestinal transit experiments were first performed (Fig. 3A, B). The results showed that, after oral administration of a consumable dye, both the distance from the pylorus to the trailing edge of the dye and the intestinal transit rate in SCI rats at 3 and 4 h were significantly lower than those in the Sham group, indicating markedly reduced gastrointestinal motility. Following BHD intervention, both parameters were significantly increased compared with the SCI group (*p* < 0.0001), suggesting that BHD effectively improves gastrointestinal motility after SCI. Histological examination further revealed severe damage in the colonic tissue of SCI rats, including thinning of the intestinal wall, epithelial cell degeneration and necrosis, and extensive inflammatory cell infiltration, whereas BHD treatment markedly alleviated these pathological changes, reduced inflammatory cell infiltration, and preserved the integrity of the intestinal wall structure (Fig. 3C). At the molecular level, the expression of representative tight junction proteins was examined (Fig. 3D). Immunofluorescence staining indicated that the expression of ZO-1 and occludin was downregulated in SCI rats, with disrupted localization, shifting from a continuous apical-lateral distribution to fragmented or abnormal patterns, suggesting impaired barrier function. BHD treatment substantially restored the expression and localization of these proteins, demonstrating its ability to stabilize tight junction structures and enhance intestinal barrier function. In summary, BHD ameliorates gastrointestinal dysfunction following SCI through the promotion of gastrointestinal motility, suppression of inflammatory responses, and restoration of tight junction protein structures, providing experimental evidence for its intestinal protective effects at functional, histological, and molecular levels.

### BHD modulates gut microbiota dysbiosis in SCI rats

**Fig. 4:**
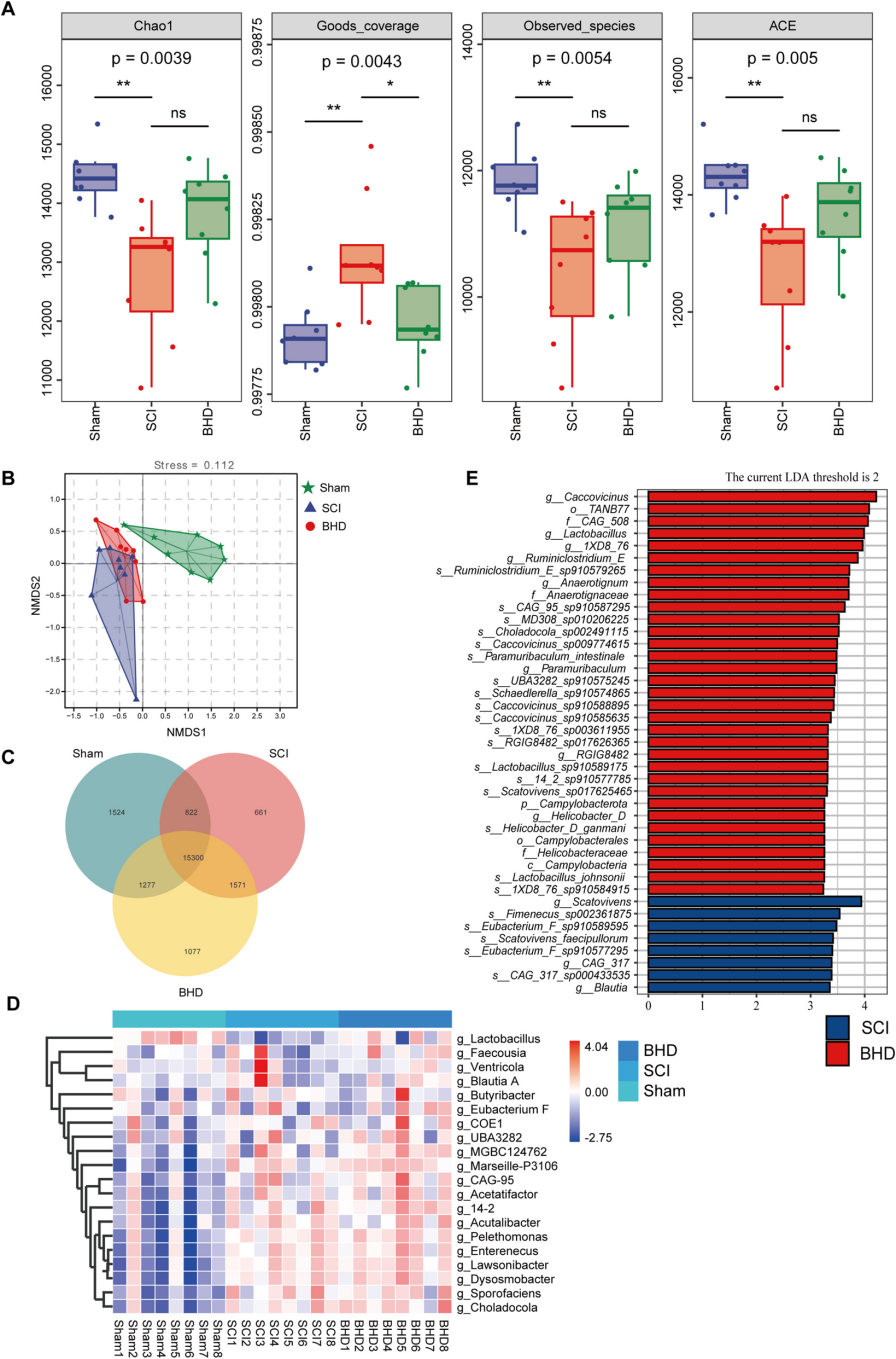
BHD modulates gut microbiota dysbiosis in rats with SCI. **A** α-diversity analysis (Chao1, ACE, Goods coverage, and Observed species) was performed using the Kruskal–Wallis rank-sum test followed by Dunn’s post hoc comparison. **B** Non-metric multidimensional scaling (NMDS) analysis based on Bray–Curtis distances revealed differences in microbial communities among groups. **C** Venn diagram illustrating differences in the number of unique species among groups. **D** Heatmap of differential genera at the genus level. **E** LEfSe analysis (P < 0.05, LDA > 2) showing significantly enriched differential taxa in each group. Data are presented as mean ± SD (n = 8). ns: no significance; *p < 0.05, **p < 0.01, ***p < 0.001, ****p < 0.0001

To evaluate the effect of BHD on the gut microbiota composition in SCI rats, fecal samples were subjected to metagenomic sequencing. Rarefaction curves, species accumulation curves, and species abundance ranking curves indicated sufficient sequencing depth (Supplementary Fig. 2). α-diversity analysis revealed a significant reduction in species richness in the SCI group (Chao1, ACE, and Observed species all decreased, all *p* < 0.01), This reduction was not significantly reversed by BHD treatment (Fig. 4A). β-diversity analysis using NMDS based on Bray–Curtis distances showed a clear separation of community structures between the Sham and SCI groups, with the BHD group positioned between them, suggesting a modulatory effect of BHD on gut microbiota composition (Fig. 4B). Regarding community structure, Venn diagram analysis demonstrated a decrease in unique microbial species in the SCI group, whereas the BHD group exhibited an increase in unique species (Fig. 4C). This observation, consistent with the higher Chao1 index shown in Fig. 4A, suggests that BHD can ameliorate microbiota loss. At the genus level, differential heatmap analysis revealed that the relative abundance of *Lactobacillus* differed significantly among groups and served as a key discriminative genus distinguishing the BHD group from the SCI group (Fig. 4D). Further LEfSe analysis (*p* < 0.05, LDA > 2) not only confirmed the differential abundance of *Lactobacillus* but also identified specific species under this genus (e.g., *Lactobacillus sp910589175* and *Lactobacillus johnsonii* [*L. johnsonii*]) as being significantly enriched in the BHD group (Fig. 4E). These findings indicate that BHD intervention not only promotes the recovery of probiotics at the genus level but also selectively enhances the colonization of specific probiotic species, further supporting its role in ameliorating SCI-induced dysbiosis through modulation of gut microbiota.

### BHD-FMT improves motor function, suppresses spinal inflammation, and promotes neural regeneration after SCI

**Fig. 5:**
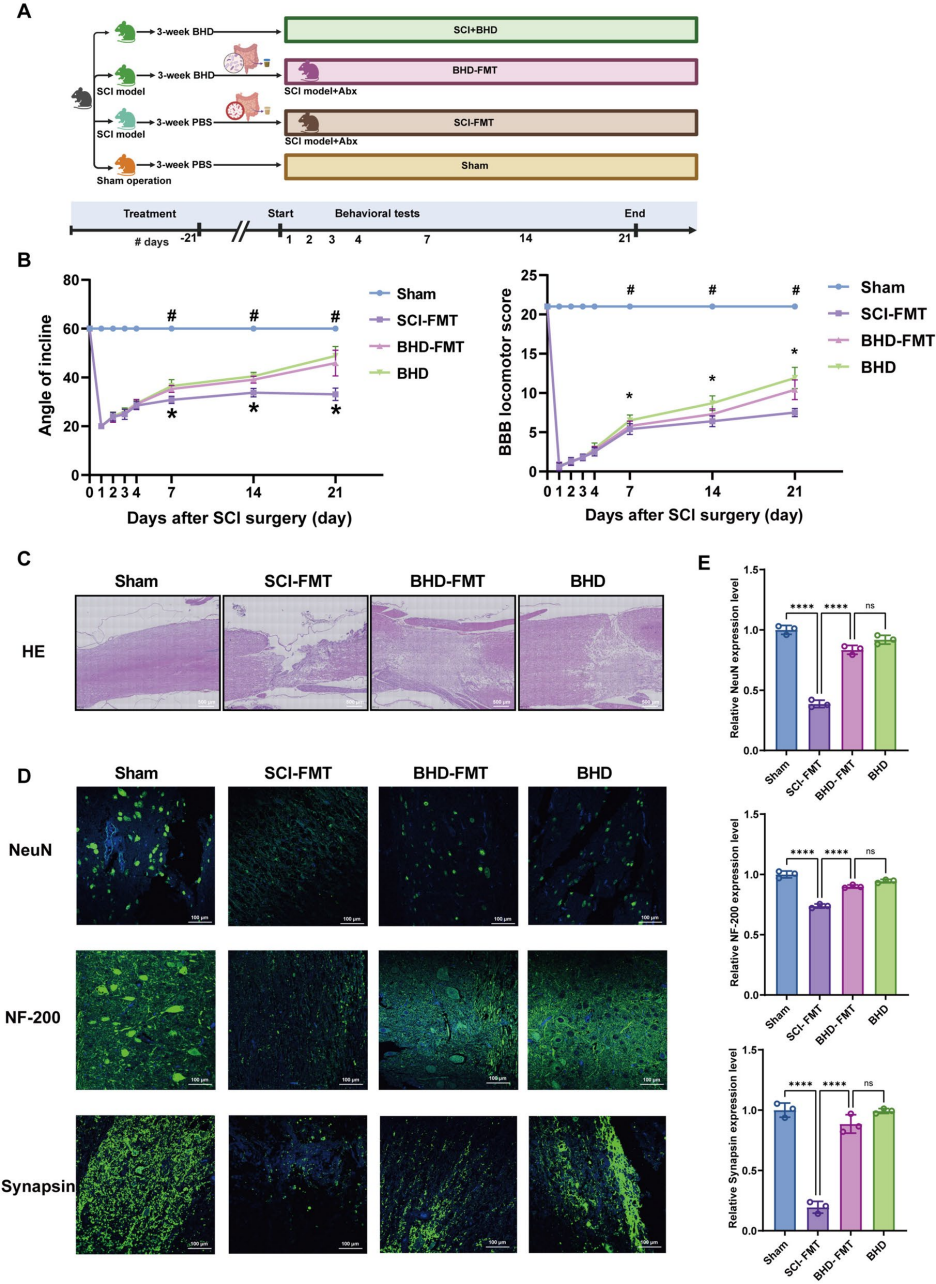
BHD-FMT improves motor function and promotes neural repair in rats with SCI. **A** Schematic of the fecal microbiota transplantation experimental procedure and group design. **B** Motor function assessed by inclined plane test and BBB scoring at indicated days post-injury (n = 10). #, Sham vs. SCI-FMT; *, SCI-FMT vs. BHD-FMT (*p* < 0.05). **C** H&E staining showing spinal cord tissue structure and inflammatory infiltration (n = 3). **D** Immunofluorescence staining of NeuN, Synapsin (SYN), and NF-200 to evaluate neuronal survival, synaptic preservation, and axonal regeneration (n = 3). **E** Quantitative analysis of NeuN-positive cells, Synapsin, and NF-200 expression across the four groups (n = 3). Data are presented as mean ± SD. Differences among multiple groups were analyzed by one-way ANOVA followed by Tukey’s post hoc test. ns: no significance; *p < 0.05, **p < 0.01, ***p < 0.001, ****p < 0.0001

To investigate the contribution of the gut microbiota to the neuroprotective effects of BHD, the fecal microbiota from SCI donors or BHD-treated donors were transplanted into SCI recipient rats (designated as SCI-FMT and BHD-FMT, respectively) (Fig. 5A). Behavioral assessments revealed that, from day 7 post-surgery, both BBB scores and inclined plane performance were significantly greater in the BHD-FMT group than in the SCI-FMT group (*p* < 0.01), with improvement levels comparable to those observed in the direct BHD administration group, suggesting that the neuroprotective effects of BHD could be partially recapitulated through microbiota transfer (Fig. 5B). Histological analysis revealed that the spinal cord lesions in the SCI-FMT group were characterized by extensive structural disruption and massive inflammatory infiltration, whereas both the BHD-FMT and BHD groups exhibited evident signs of repair, including smaller lesion areas and markedly reduced inflammatory cell infiltration (Fig. 5C, Supplementary Fig. 1B). Immunofluorescence analysis revealed that SCI-FMT resulted in a marked loss of NeuN-positive neurons, decreased SYN expression, and weakened NF-200 signals, whereas these alterations were significantly reversed in the BHD-FMT group (*p* < 0.0001) (Fig. 5D). Quantitative analyses, including NeuN-positive cell density, SYN fluorescence intensity, and NF-200-positive fiber area (Fig. 5E), consistently indicated that BHD-FMT could partially reproduce the neuroprotective effects of BHD in the absence of direct pharmacological intervention. Collectively, FMT from BHD-treated donors partially reproduced the effects of BHD on motor recovery, inflammation suppression, and neural repair in recipient rats, supporting the critical mediating role of the gut microbiota in the therapeutic actions of BHD.

### BHD-FMT ameliorates gastrointestinal dysfunction, suppresses intestinal inflammation, and enhances intestinal barrier function following SCI

**Fig. 6:**
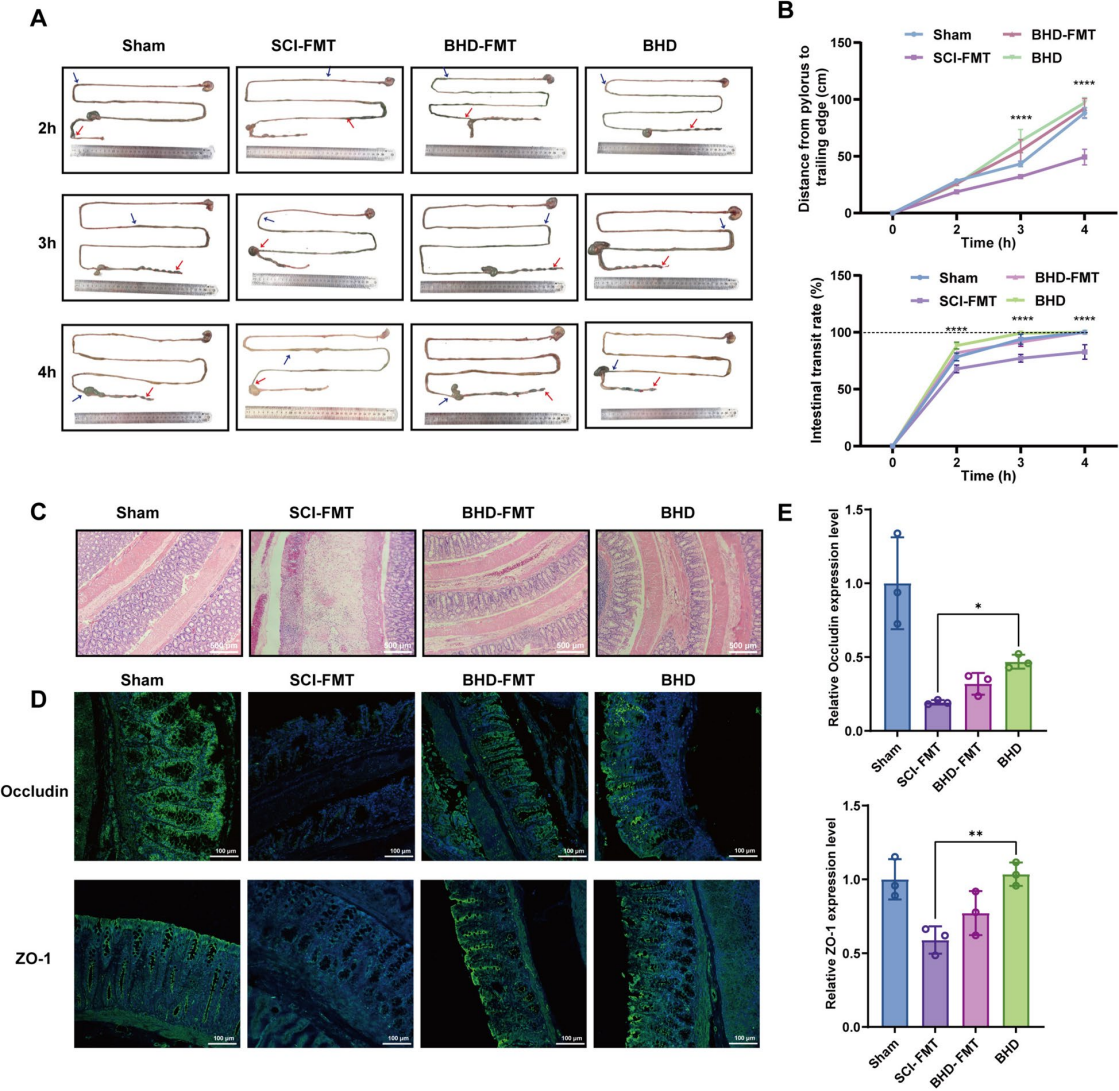
BHD-FMT ameliorates gastrointestinal dysfunction and enhances intestinal barrier integrity in rats with SCI. **A** Representative images of gastrointestinal dye distribution at indicated time points post-gavage (2 h, 3 h, and 4 h). Blue and red arrows indicate the proximal and distal ends of the intestinal dye front, respectively. **B** Quantitative analysis of gastrointestinal motility in rats at 2, 3, and 4 h after oral administration of edible dye. Parameters included the distance from the pylorus to the trailing edge of the dye and the intestinal transit rate (%). **C** H&E staining of the colon showing tissue structure and inflammatory infiltration (n = 3). **D**, **E** Immunofluorescence staining of ZO-1 and Occludin to evaluate expression and localization of tight junction proteins (n = 3). Data are presented as mean ± SD. Differences among multiple groups were analyzed by one-way ANOVA followed by Tukey’s post hoc test. ns: no significance; *p < 0.05, **p < 0.01, ***p < 0.001, ****p < 0.0001

To evaluate the mediating role of the gut microbiota in the intestinal protective effect of BHD, the same FMT protocol was employed to assess gastrointestinal motility and intestinal barrier function. The oral dye transit test revealed that both the distance from the pylorus to the trailing edge of the dye and the intestinal transit rate in the SCI-FMT group were significantly reduced compared with those in the BHD-FMT group at 3 h post-injury, while the BHD-FMT group exhibited values comparable to those observed in the direct BHD gavage group (Fig. 6A, B). To further quantify the functional recovery of the gut, we assessed fecal water content and Bristol Stool Form Scale (BSFS) scores on day 21 post-SCI. Consistent with the transit assay, both BHD and BHD-FMT treatments improved these parameters compared with the SCI-FMT group. However, direct BHD administration resulted in significantly higher fecal water content and more favorable BSFS scores than BHD-FMT (Supplementary Fig. 3), indicating a more robust restoration of intestinal homeostasis by the full decoction. Histopathological analysis of the intestinal tissue was further performed. H&E staining demonstrated that the SCI-FMT group exhibited severe structural disruption of the colon, including mucosal degeneration, epithelial cell necrosis, and extensive inflammatory infiltration (Fig. 6C). In contrast, the BHD-FMT group showed marked restoration of colonic architecture, improved mucosal integrity, and decreased inflammatory infiltration, indicating that FMT alleviates intestinal inflammatory injury following SCI. To assess intestinal barrier function, the expression and subcellular localization of the tight junction proteins ZO-1 and Occludin were analyzed (Fig. 6D, E). In the SCI-FMT group, both proteins exhibited reduced expression and disorganized localization, characterized by fragmented or discontinuous distribution. In the BHD-FMT group, ZO-1 and Occludin expression was restored, displaying a continuous apical-lateral distribution pattern, suggesting that the intestinal barrier structure was reconstructed. Collectively, these findings demonstrate that FMT derived from BHD partially reproduces the protective effects of direct BHD administration on gastrointestinal motility, intestinal inflammation, and barrier restoration, supporting the gut microbiota as a crucial mediator of its therapeutic efficacy.

### BHD-FMT significantly reshapes the dysbiotic gut microbiota after SCI

**Fig. 7:**
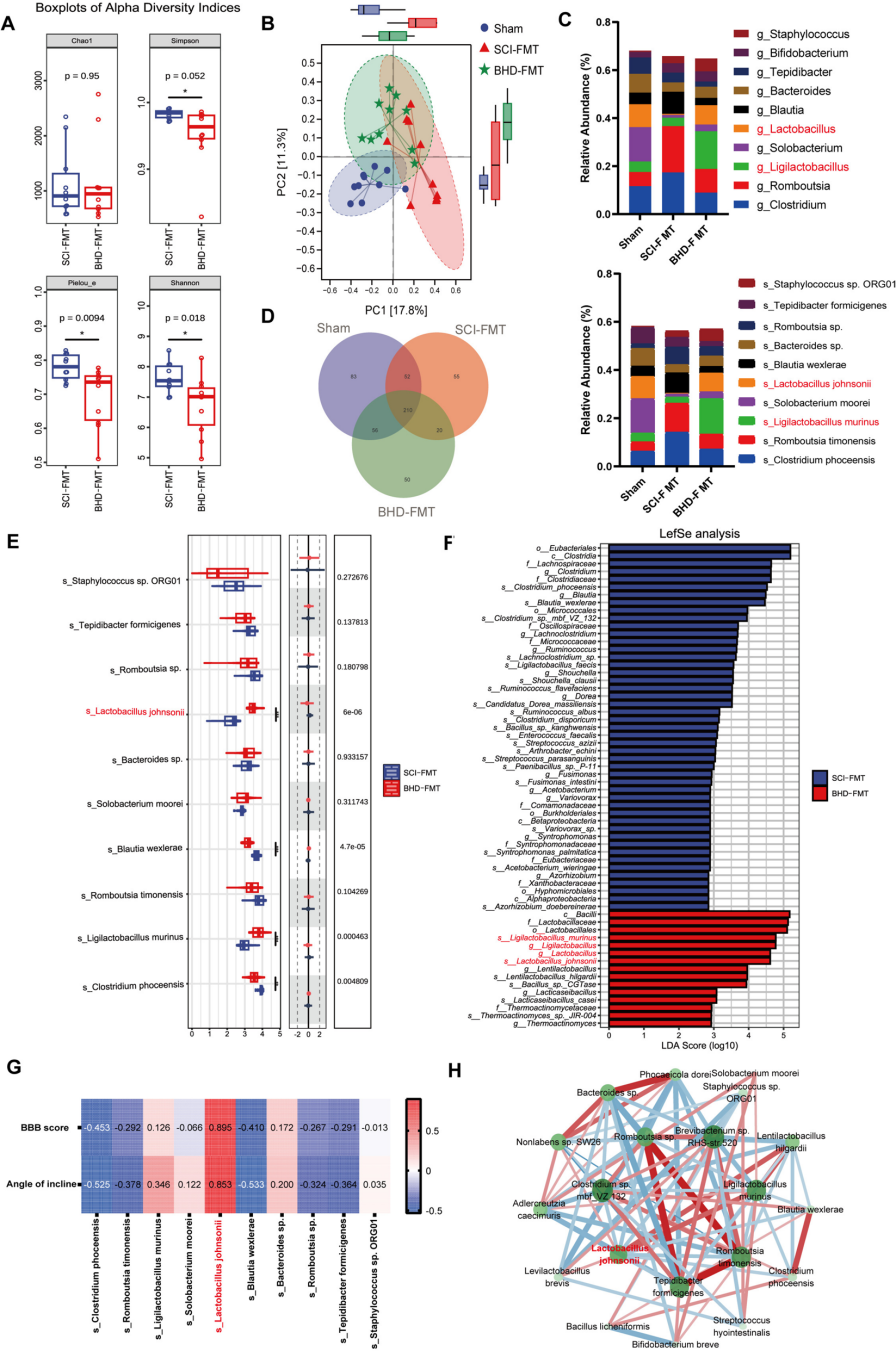
BHD-FMT reshapes gut microbiota composition in rats with SCI. **A** α-diversity analysis including Chao, Simpson, Pielou_e, and Shannon indices. **B** β-diversity analysis using principal coordinates analysis (PCoA) to reveal differences in community structure among groups. **C** Taxonomic analysis at the genus and species levels showing changes in the abundance of major microbial taxa. **D** Venn diagram illustrating differences in the number of unique ASVs among groups. **E** Species-level differential analysis of microbial taxa. **F** LEfSe analysis (P < 0.05, LDA > 5) showing significantly enriched differential taxa and key probiotic species among groups. **G** Spearman correlation analysis displaying associations between key microbial taxa and motor function indices. **H** Ecological network analysis illustrating interactions between key probiotic species and potential pathogenic bacteria. Data are presented as mean ± SD (n = 10). Differences in α-diversity indices among groups were analyzed by the Kruskal–Wallis test followed by Dunn’s post hoc test. PCoA was based on Bray–Curtis distances and group differences were tested by PERMANOVA. Correlations in (**G**) were assessed by Spearman’s rank correlation test. ns: no significance; *p < 0.05, **p < 0.01, ***p < 0.001, ****p < 0.0001

Considering that BHD-FMT alleviated intestinal dysfunction following SCI, 16S rDNA (V3–V4 region) sequencing of fecal samples was conducted to further investigate its modulatory effects on the gut microbial community structure. α-Diversity analyses (Chao, Simpson, Pielou_e, and Shannon indices) revealed that the BHD-FMT group did not display a significant increase but rather a moderate reduction in α-diversity (Fig. 7A). This finding suggests that the therapeutic effects of BHD-FMT are not primarily mediated by an overall increase in microbial diversity, but are more likely dependent on the colonization and enrichment of specific functional taxa. β-Diversity analysis (PCoA) demonstrated that BHD-FMT significantly reshaped the gut microbiota composition after SCI, with its community positioned intermediate between the Sham and SCI-FMT groups (Fig. 7B), indicating a partial restoration of the global microbial structure. Taxonomic profiling revealed that although differences at the phylum level were not significant (Supplementary Fig. 1), pronounced alterations were observed at the genus and species levels (Fig. 7C). BHD-FMT significantly restored the reduced abundances of *Ligilactobacillus* and *Lactobacillus* following SCI, while suppressing the aberrant overgrowth of *Clostridium* and *Romboutsia*. At the species level, *L. murinus* and *L. johnsonii* were markedly enriched in the BHD-FMT group. Regarding community composition, the Venn diagram illustrated that SCI-FMT induced a large number of unique ASVs, whereas the BHD-FMT group exhibited a markedly reduced number of unique ASVs (Fig. 7D), implying that intestinal microecological reconstruction may occur via modulation of key taxa. The Student’s t-test, combined with multiple comparison correction, was applied to analyze differences in taxonomic abundances between the two groups (Fig. 7E). Among the top 10 dominant species, *L. johnsonii* exhibited the most significant intergroup difference (*p* < 0.001). LEfSe analysis further confirmed that *Bacilli*, *Lactobacillales*, *Lactobacillaceae*, *Ligilactobacillus*, *Lactobacillus*, and their representative species *L. murinus* and *L. johnsonii* were significantly enriched in the BHD-FMT group (*p* < 0.05, LDA > 5; Fig. 7F). Spearman’s correlation analysis revealed that the relative abundance of *L. johnsonii* was positively correlated with both BBB scores and inclined plane performance (*p* < 0.05, Fig. 7G), suggesting its potential functional relevance. Subsequently, an ecological co-occurrence network was constructed based on the differential taxa identified between the BHD-FMT and SCI-FMT groups (Fig. 7H). The results indicated that *L. johnsonii* was negatively associated with multiple potential pathogens, suggesting that it may inhibit pathogenic overgrowth through ecological antagonism, thereby maintaining intestinal homeostasis. In summary, BHD-FMT reshapes the intestinal microecology at the genus and species levels by enriching key probiotic taxa—particularly *L. johnsonii*—which are statistically associated with functional recovery, suggesting that these taxa represent potential core strains mediating the therapeutic efficacy of BHD-FMT.

### BHD-FMT upregulates gut-derived tryptophan metabolites

**Fig. 8:**
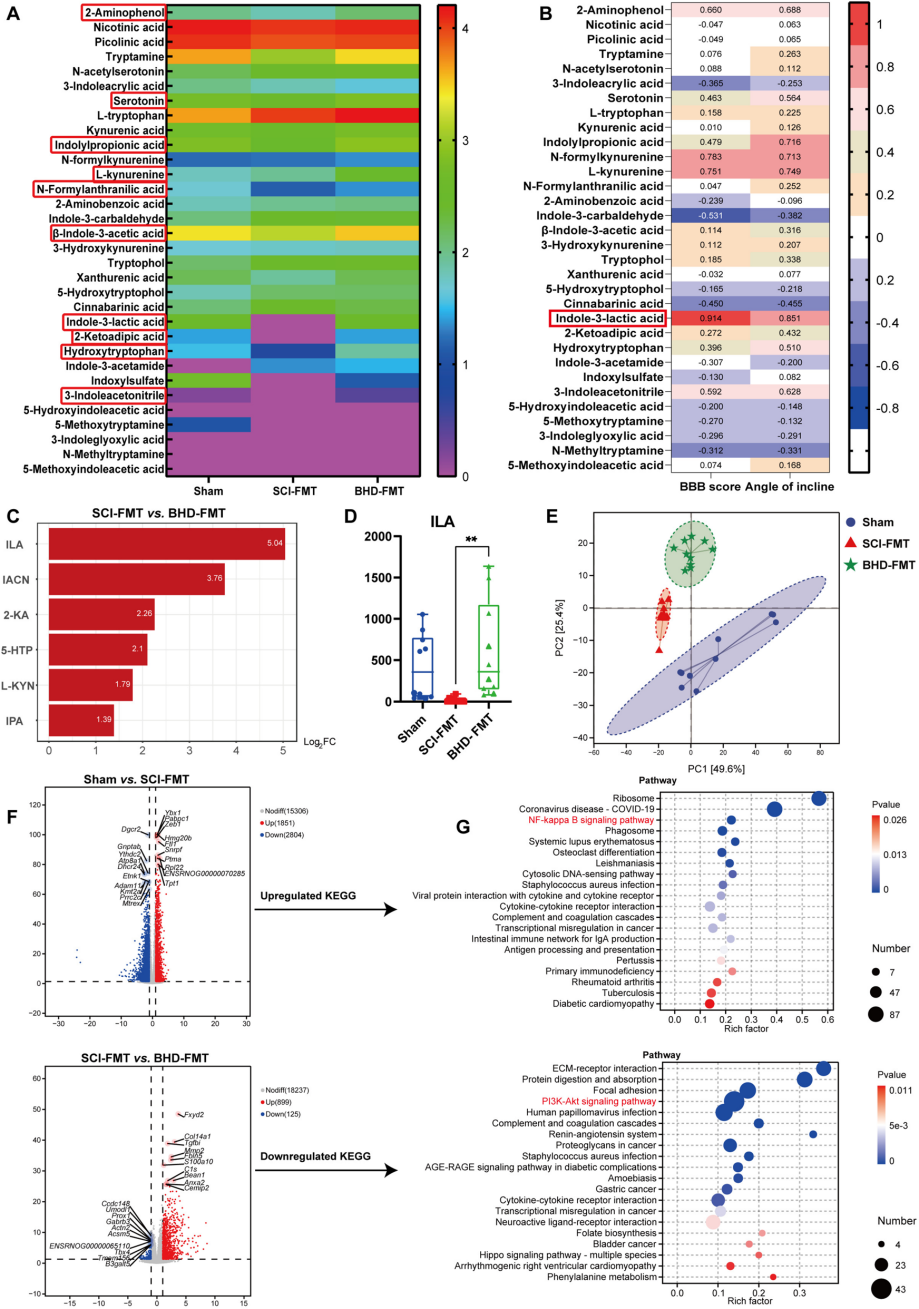
BHD-FMT upregulates tryptophan metabolites and activates the spinal PI3K/Akt signaling pathway. **A** Heatmap of targeted tryptophan metabolomics showing the distribution of 34 metabolites across three groups; red labels indicate 10 significantly different metabolites between the BHD-FMT and SCI-FMT groups (*p* < 0.05). **B** Spearman correlation analysis displaying the association between ILA levels and motor function scores. **C** Differential metabolite analysis (VIP > 1, fold change ≥ 2 or ≤ 0.5) showing significant upregulation of ILA, IACN, 2-KA, 5-HTP, L-KYN, and IPA in the BHD-FMT group. **D** Levels of ILA across the three groups. **E** PCA of spinal cord transcriptomics. **F** Volcano plot of differentially expressed genes showing significantly upregulated and downregulated genes among groups. **G** KEGG pathway enrichment analysis, with bubble size representing the number of metabolites and color indicating the P value. Data are presented as mean ± SD (n = 10). Differences in metabolite levels and IPA among groups were analyzed by one-way ANOVA followed by Tukey’s post hoc test. The correlation in (B) was assessed by Spearman’s rank correlation test. ns: no significance; *p < 0.05, **p < 0.01, ***p < 0.001, ****p < 0.0001

Alterations in the gut microbiota composition are typically accompanied by changes in the metabolic profile, particularly in tryptophan metabolites associated with neuroregulation [48]. To investigate whether BHD-FMT affects functional metabolites, a targeted tryptophan metabolomics analysis was performed on fecal samples from each group, with particular focus on indole-derived tryptophan metabolites associated with the gut microbiota. A total of 34 metabolites were identified, among which 20 were indole derivatives (Supplementary Table 2). A heatmap illustrated the distribution profiles of the 34 metabolites among the three groups, revealing ten significantly altered metabolites between the BHD-FMT and SCI-FMT groups (*p* < 0.05; Fig. 8A). Further analysis indicated that in the BHD-FMT group, the levels of ILA, IACN, 2-KA, 5-HTP, L-KYN, and IPA were significantly increased, with ILA showing the most prominent elevation (fold change ≥ 2, *p* < 0.05), suggesting that it may play a crucial role in the repair process following SCI (Fig. 8C and D). Spearman’s correlation analysis revealed a significant positive association between ILA levels and both the BBB and inclined plane test scores (r > 0.8, *p* < 0.05; Fig. 8B), suggesting a potential link between ILA and motor function recovery.

### BHD-FMT activates the PI3K/Akt signaling pathway

**Fig. 9:**
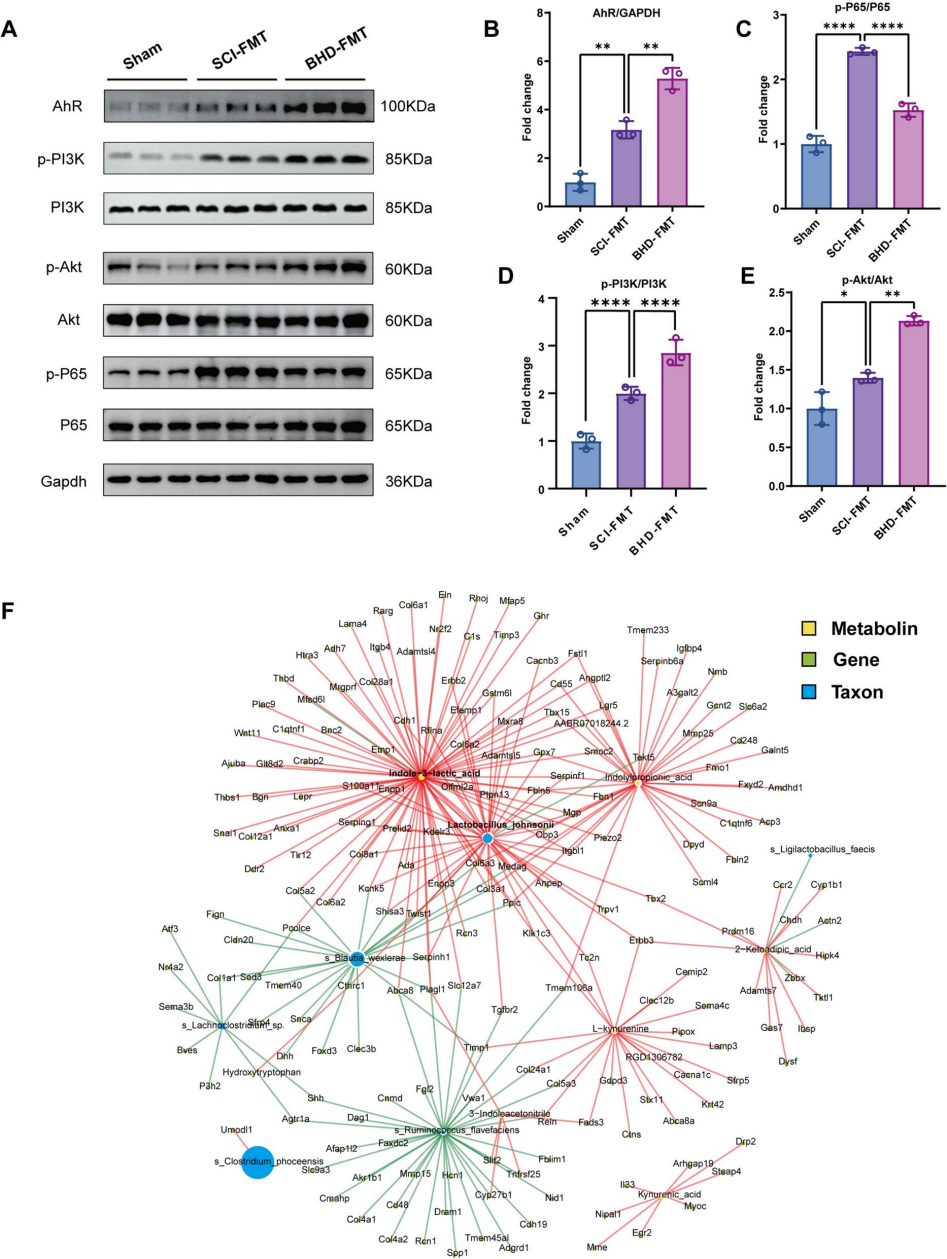
Multi-omics integration reveals a “*L. johnsonii*–ILA–PI3K/Akt”-mediated gut–spinal cord axis signaling network. **A** Western blot analysis showing AhR, p-PI3K, PI3K, p-Akt, Akt, p-P65, and P65 expression in the Sham, SCI-FMT, and BHD-FMT groups, with GAPDH used as a loading control. **B**–**E** Quantitative analysis of AhR/GAPDH, p-P65/P65, p-PI3K/PI3K, and p-Akt/Akt expression (n = 3). Data are presented as mean ± SD. Differences among groups were analyzed by one-way ANOVA followed by Tukey’s post hoc test. ns: no significance; *p < 0.05, **p < 0.01, ***p < 0.001, ****p < 0.0001. **F** Multi-omics correlation network visualized using Cytoscape, comprising six key microbial taxa, seven metabolites, and 193 differentially expressed genes. Nodes represent key microbes (blue), metabolites (green), and genes (yellow), while edges indicate significant correlations (Spearman |r|> 0.8, *p* < 0.05), with red lines representing positive correlations and green lines representing negative correlations

Gut-derived metabolites, particularly tryptophan and its indole derivatives, may influence central nervous system function via the blood–spinal cord barrier. To identify the key spinal cord signaling pathways mediated by BHD-FMT, transcriptomic analysis was performed on spinal cord tissues from each group. Principal component analysis (PCA) revealed clear separation among the Sham, SCI-FMT, and BHD-FMT groups in the global gene expression profiles (Fig. 8E), indicating that the treatment intervention markedly altered transcriptional patterns. Differential gene expression analysis (fold change > 2, *p* < 0.05) showed that, compared with the Sham group, the SCI-FMT group had 1,851 upregulated and 2,804 downregulated genes; in contrast, the BHD-FMT group exhibited 885 upregulated and 96 downregulated genes compared with the SCI-FMT group (Fig. 8F). KEGG pathway enrichment analysis (Fig. 8G) indicated that inflammatory signaling pathways, such as NF-κB, were significantly upregulated in the SCI-FMT group, reflecting pronounced SCI-induced inflammatory responses. In contrast, BHD-FMT markedly activated multiple pathways associated with tissue repair and neuroprotection, including ECM–receptor interaction, protein digestion and absorption, focal adhesion, and the PI3K/Akt signaling pathway. Notably, the PI3K/Akt pathway contained the highest number of enriched genes, suggesting that it may serve as a key pathway mediating BHD-FMT-induced neuroprotection. Western blot analysis further confirmed that BHD-FMT reduced SCI-FMT-induced p-p65 expression while enhancing p-Akt expression (Fig. 9A–D). These findings were consistent with the RNA-seq results, supporting its role as a potential central signaling regulator in this study.

### Multi-omics integration reveals a potential “*L. johnsonii*–ILA–PI3K/Akt” gut–spinal cord regulatory network

Based on the aforementioned analyses of gut microbiota, metabolites, and spinal cord transcriptomes, multi-omics integration was further performed to construct a gut–spinal cord regulatory network. Firstly, significantly differential microbial taxa, metabolites, and transcriptomic genes were identified between the BHD-FMT and SCI-FMT groups, and strong associations were determined through Spearman correlation analysis (|r|> 0.8, *p* < 0.05). The network was then visualized using Cytoscape v3.10.2 (Fig. 9B). The resulting network comprised 6 key microbial taxa, 7 differential metabolites, and 193 significantly altered genes, exhibiting a complex pattern of microbe–metabolite–gene interactions. Network analysis indicated that ILA exhibited the highest degree among metabolite nodes and was significantly positively correlated with *L. johnsonii*. Simultaneously, *L. johnsonii* also displayed high connectivity among microbial nodes, suggesting that both occupy central positions in gut–spinal cord interactions. Further analysis revealed that among 34 genes positively correlated with ILA, 6 genes were significantly enriched in the PI3K/Akt signaling pathway (e.g., *Col6a1, Col6a2, Col6a3, Lama4, Itgb4,* and *Ghr*). These genes are involved in extracellular matrix remodeling and cell survival, which are closely associated with neural repair processes. Collectively, these integrated multi-omics correlations suggest that the “*L. johnsonii*–ILA–PI3K/Akt” axis may constitute a key signaling pathway mediating the effects of BHD-FMT.

### BHD-FMT neuroprotective effects depend on AhR mediation

**Fig. 10:**
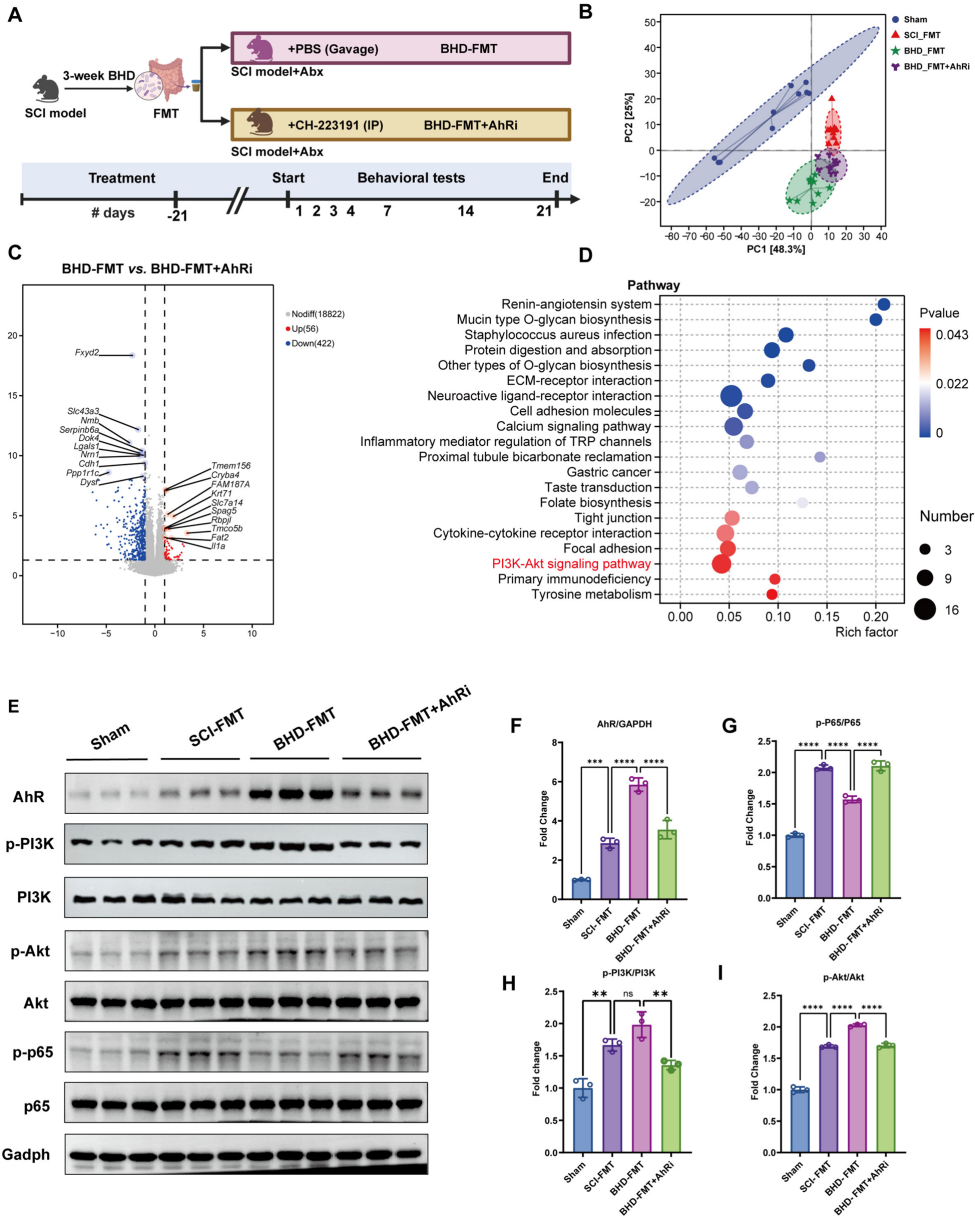
AhR inhibition attenuates BHD-FMT-mediated activation of the PI3K/Akt signaling pathway. **A** Schematic of the experimental design: BHD-FMT combined with an AhR antagonist. **B** PCA of spinal cord transcriptomics (n = 10). **C** Volcano plot of differentially expressed genes showing significantly upregulated and downregulated genes among groups (n = 10). **D** KEGG pathway enrichment analysis, with bubble size representing the number of metabolites and color indicating the P value (n = 10). **E** Western blot analysis showing AhR, p-PI3K, PI3K, p-Akt, Akt, p-P65, and P65 expression in the Sham, SCI-FMT, BHD-FMT, and BHD-FMT + AhRi groups, with GAPDH used as a loading control. **F**–**I** Quantitative analysis of AhR/GAPDH, p-P65/P65, p-PI3K/PI3K, and p-Akt/Akt expression (n = 3). Data are presented as mean ± SD. Differences among multiple groups were analyzed by one-way ANOVA followed by Tukey’s post hoc test. ns: no significance; *p < 0.05, **p < 0.01, ***p < 0.001, ****p < 0.0001

Tryptophan metabolites are considered important endogenous AhR agonists, capable of mediating immune regulation and barrier repair [28]. Western blot analysis confirmed that AhR protein expression was significantly upregulated by BHD-FMT (*p* < 0.01, Fig. 9A, B). In addition, BHD-FMT significantly increased the p-PI3K/PI3K and p-Akt/Akt ratios while reducing p-P65/P65 levels compared with the SCI-FMT group (Fig. 9A, C–E), providing protein-level support for activation of the PI3K/Akt pathway and suppression of NF-κB signaling. Considering that AhR can regulate the PI3K/Akt signaling axis via downstream transcriptional mechanisms, it was hypothesized that BHD-FMT may exert neuroprotective effects through a “tryptophan–AhR–PI3K/Akt” metabolic–signaling axis. To verify the requirement of AhR for BHD-FMT efficacy, a selective AhR antagonist (CH-223191) was administered in combination with BHD-FMT, and outcomes were compared with the BHD-FMT control group (Fig. 10A). PCA analysis revealed that the spinal cord transcriptome of the AhR-inhibited group deviated markedly from the BHD-FMT group and approximated that of the SCI-FMT group (Fig. 10B). Differential gene analysis indicated that 48 genes were upregulated and 383 genes were downregulated in the AhR-inhibited group relative to the BHD-FMT group (Fig. 10C). KEGG enrichment analysis confirmed that PI3K/Akt pathway enrichment was significantly suppressed in the BHD-FMT + AhRi group (Fig. 10D). At the protein level, Western blot analysis showed that AhR expression was markedly downregulated in the BHD-FMT + AhRi group, accompanied by significantly reduced p-PI3K/PI3K and p-Akt/Akt ratios, as well as increased p-P65/P65 levels, compared with the BHD-FMT group (Fig. 10E–I), indicating effective blockade of the AhR–PI3K/Akt signaling pathway.

**Fig. 11:**
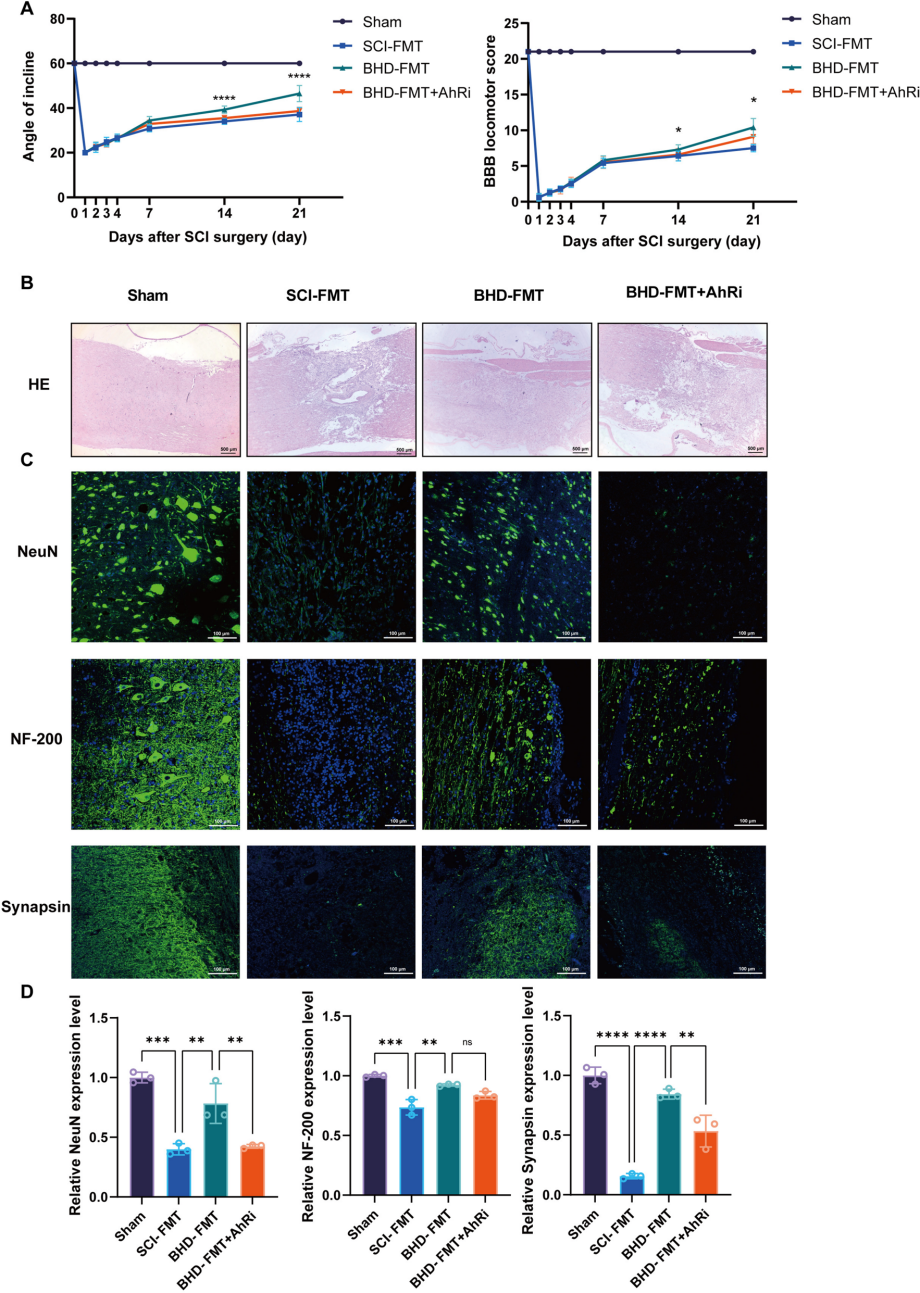
AhR inhibition blocks the spinal protective effects mediated by BHD-FMT. **A** Inclined plane test and BBB assessments evaluating motor function at different time points post-injury (1, 2, 3, 4, 7, 14, and 21 days). Data are presented as mean ± SD (n = 10). *, BHD-FMT vs. BHD-FMT + AhRi (*p* < 0.05). Behavioral scores were analyzed by two-way repeated measures ANOVA followed by Sidak’s post hoc test. **B** H&E staining showing spinal cord tissue structure and inflammatory infiltration (n = 3). **C** Immunofluorescence staining for NeuN, SYN, and NF-200 to assess neuronal survival, synaptic protection, and axonal regeneration (n = 3). **D** Quantitative analysis of NeuN-positive cells, Synapsin, and NF-200 expression across the three groups (n = 3). Data are presented as mean ± SD. Differences among multiple groups were analyzed by one-way ANOVA followed by Tukey’s post hoc test. ns: no significance; *p < 0.05, **p < 0.01, ***p < 0.001, ****p < 0.0001

To determine whether the neuroprotective effects of BHD-FMT depend on AhR, the therapeutic outcomes in SCI rats were assessed following AhRi administration. Functional assessments revealed that BBB scores in the BHD-FMT + AhRi group were significantly lower than those in the BHD-FMT group from day 14 onward, and the inclined plane test on day 21 also indicated impaired functional recovery (Fig. 11A). Histological and immunofluorescence analyses demonstrated increased inflammatory cell infiltration in the spinal cord of the AhR-inhibited group (Fig. 11B). Quantitative analysis further revealed that AhR inhibition completely reversed the effect of BHD-FMT on the reduction of spinal cord lesion area (Supplementary Fig.  1C), which was also accompanied by loss of NeuN-positive neurons, decreased SYN expression, and reduced NF-200 signals (Fig. 11C, D). These findings suggest that AhR inhibition partially abrogates the molecular and functional effects of BHD-FMT, supporting a critical role for AhR in the BHD-mediated gut–spinal cord axis.

## Discussion

In recent years, the gut–spinal cord axis, as an important physiological pathway connecting the gut and the central nervous system, has increasingly been recognized as a key mechanistic basis underlying SCI repair [8, 49]. Several studies have suggested that modulation of the gut microbiota can enhance neuronal protection, alleviate central inflammation, and even promote motor function recovery [50–53]. BHD, a classical prescription for treating injuries of the central nervous system, has shown potential in SCI therapy, yet its underlying mechanisms—particularly whether they involve the recently highlighted gut–spinal cord axis—remain unclear. In this study, a series of functional experiments combined with multi-omics analyses were conducted, demonstrating for the first time that BHD promotes SCI repair by reshaping the gut microbiota, regulating its metabolites, and activating downstream signaling pathways via the gut–spinal cord axis. Accordingly, a *L. johnsonii*–ILA–AhR–PI3K/Akt signaling network was constructed, revealing a novel mechanism by which BHD mediates SCI intervention through the gut–spinal cord axis.

First, this study confirmed the therapeutic effects of BHD on SCI. Consistent with previous reports [54], oral administration of BHD significantly promoted motor function recovery following SCI. Histological analysis further demonstrated that this functional improvement was accompanied by multiple pathological improvements in spinal cord tissue, including attenuated inflammatory responses and markedly increased expression of neuroregeneration-related markers. Notably, secondary neurogenic bowel dysfunction (NBD) has been widely recognized as a major factor limiting neural repair and functional recovery after SCI, typically characterized by reduced intestinal motility, disruption of the intestinal barrier, and increased mucosal permeability [55, 56]. Among these manifestations, intestinal barrier injury—characterized by the loss of tight junction proteins and barrier leakage—has been considered a major driver of microbial and metabolite translocation that induces secondary central inflammation [57]. Our experimental results revealed that BHD markedly alleviated NBD after SCI, as evidenced by enhanced intestinal motility, restoration of barrier integrity (increased Occludin and ZO-1 expression), and reduced intestinal inflammation. Previous studies have indicated that the integrity and inflammatory status of the intestinal barrier are critical prerequisites for maintaining the structural and functional homeostasis of the gut microbiota, and their impairment is often accompanied by dysbiosis and metabolic disturbances [58, 59]. Our findings are highly consistent with these observations, suggesting that BHD may promote microbial reconstruction and functional recovery by repairing the intestinal barrier and alleviating the “leaky gut” condition.

Metagenomic sequencing revealed that SCI markedly induced gut microbiota dysbiosis, characterized by a global decline in microbial diversity and an abnormal increase in the relative abundance of *Bacteroidetes* and its subordinate taxa. Previous studies have reported that dysregulation of Bacteroidetes is associated with increased endotoxin release, which may exacerbate neurodegenerative processes by activating microglia [60]. Our study demonstrated that BHD not only restored microbial diversity but also significantly increased the abundance of *Firmicutes*, with a particularly prominent enrichment of *Lactobacillus*. It has been reported that *Lactobacillus* promotes mucus layer formation and upregulates tight junction proteins, thereby reducing intestinal permeability and preventing the translocation of endotoxins and pro-inflammatory cytokines from the gut into the systemic circulation [61, 62]. Further component analysis revealed that BHD contains multiple bioactive compounds, including paeoniflorin, ferulic acid, and astragaloside IV. Studies have shown that paeoniflorin can coordinately modulate the intestinal immune microenvironment, thereby enhancing the colonization ability of *Lactobacillus* [63, 64]. Ferulic acid, a phenolic compound metabolizable by *Lactobacillus*, has been shown to be converted into multiple metabolites during bacterial growth, suggesting that it may serve as a carbon source to promote bacterial proliferation [65]. Moreover, astragaloside IV indirectly supports the growth of *Lactobacillus* by modulating the intestinal microecology, as evidenced by multiple animal studies showing increased *Lactobacillus* abundance in treated groups [66–69]. These synergistic effects collectively explain the mechanism by which BHD mediates the remodeling of the gut microbiota. To directly verify the causal role of the gut microbiota in the therapeutic effects of BHD, a FMT experiment was performed. Transplantation of fecal microbiota from BHD-treated donor rats into SCI recipient rats reproduced most of the beneficial effects observed with direct BHD administration, including improved motor function, suppressed spinal inflammation, enhanced neuroregeneration, and ameliorated intestinal function. In contrast, transplantation of microbiota from SCI donor rats failed to exert such effects. This pivotal experiment clearly demonstrated that the therapeutic efficacy of BHD largely depends on its modulation of the host gut microbial ecosystem rather than solely on the direct effects of its chemical constituents, providing a modern biological interpretation for the multi-component, multi-target, and holistic regulatory characteristics of traditional Chinese medicine formulas. Building upon this, a nuanced comparison between the two interventions provided further insight. Although BHD-FMT effectively transferred a therapeutic gut microbiota capable of mediating significant functional and structural recovery, direct administration of the full BHD decoction consistently yielded superior outcomes across both neurological and gastrointestinal parameters. This suggests that the full therapeutic spectrum of BHD includes both a transferable, microbiota-mediated component and additional, direct protective effects of its herbal constituents on intestinal tissue. Thus, the FMT experiment definitively isolates a central mechanistic pathway, while the direct comparison highlights the integrative, synergistic pharmacology that contributes to the holistic decoction’s superior tissue repair outcomes.

After establishing the central role of gut microbiota reconstruction, this study further elucidated the regulatory mechanisms of BHD-FMT. Interestingly, distinct effects on microbial α-diversity were observed between direct BHD gavage and BHD-FMT: BHD gavage significantly increased the α-diversity of SCI rats, whereas BHD-FMT failed to restore α-diversity but notably altered β-diversity. Despite these differences in overall microbial diversity, both treatments effectively improved motor function and intestinal barrier integrity. This difference may result from the fact that BHD gavage delivers both active compounds and fermentable substrates, thereby promoting overall microbial proliferation, whereas BHD-FMT enriches functional core probiotics and suppresses opportunistic pathogens, maintaining microecological homeostasis and achieving precise functional reconstruction. Similarly, Zhang et al. [70] reported that the therapeutic efficacy of FMT often depends on the reestablishment of specific probiotic communities rather than merely on the restoration of overall diversity. This finding supports the crucial role of core probiotics and their metabolic functions in mediating the intervention effects. Further taxonomic analysis revealed that typical Lactobacillus species, such as *L. johnsonii* and *L. murinus*, were significantly enriched in the BHD-FMT group. These strains have been widely documented to participate in multiple physiological processes, including mucosal immune regulation, tryptophan metabolism, and epithelial barrier repair [71–73]. Among them, *L. johnsonii*, as the most significantly altered functional probiotic, ranked among the top features in LEfSe and random forest analyses, and its abundance showed a significant positive correlation with behavioral parameters such as the BBB score and inclined plane test. *L. johnsonii* has been reported to promote motor function recovery in models of neurological injury and degenerative diseases via the gut–brain axis [74, 75]. Its mechanisms include attenuating secondary neuroinflammation through downregulation of pro-inflammatory cytokines and suppression of microglial and astrocytic overactivation [74, 76]. Given these neuroprotective properties, we hypothesize that the enrichment of *L. johnsonii* may constitute a key microecological mechanism underlying the improvement in motor function observed after SCI with BHD treatment.

Numerous studies have demonstrated that *Lactobacillus* can synthesize various indole derivatives through the tryptophan metabolic pathway, which play essential roles in maintaining intestinal mucosal homeostasis and immune regulation [9, 77]. Targeted tryptophan metabolomic analysis revealed that multiple indole derivatives were markedly elevated in the feces of rats in the BHD-FMT group, particularly ILA, IPA, 5-HTP, and IACN. These indole metabolites are widely recognized as endogenous agonists of the AhR, which can promote intestinal epithelial repair and enhance barrier function by inducing *IL-22* secretion, while suppressing aberrant activation of inflammatory cytokines to maintain immune homeostasis and confer neuroprotection [78–80]. Among these metabolites, ILA exhibited the most prominent upregulation and showed a significant positive correlation with behavioral indices such as the BBB score and inclined plane test, suggesting its potential pivotal role in functional recovery after SCI. Previous studies have demonstrated that microbiota-derived ILA directly modulates neuroinflammation via the AhR signaling pathway, thereby influencing neurological outcomes [81]. This mechanism has also shown therapeutic potential in models of multiple sclerosis [82], anxiety and depression [83], and Alzheimer’s disease [84]. Further microbiota–metabolite correlation analysis revealed that *L. johnsonii*, a core species significantly enriched in the BHD-FMT group, was strongly correlated with ILA levels, suggesting that it may serve as the primary producer of this metabolite, although direct in vitro verification of its ILA-producing capacity was not performed in this study. Previous evidence has also confirmed that *L. johnsonii* possesses a stronger capacity for tryptophan metabolism than other lactic acid bacteria, particularly in the production of ILA [85]. In summary, BHD-FMT may enhance tryptophan metabolism by enriching functional probiotics such as *L. johnsonii*, thereby promoting the production of indole derivatives such as ILA, activating the AhR signaling axis, and exerting dual effects on barrier protection and neural repair.

In terms of signaling pathways, tryptophan metabolites are closely associated with genes involved in neural regulation [86]. Studies have shown that microbiota-derived tryptophan metabolites can influence the central nervous system by activating the AhR in astrocytes [87, 88]. As an upstream regulatory factor, AhR can initiate the expression of multiple genes and mediate a cascade of downstream responses [89]. For instance, it plays a crucial role in regulating various signaling pathways associated with neuroinflammatory responses and neuronal regeneration [90–92]. RNA-seq and Western blot analyses revealed that SCI-FMT significantly enriched genes associated with the NF-κB signaling pathway, which is likely closely related to post-SCI neuroinflammation [93]. Meanwhile, BHD-FMT treatment markedly upregulated the expression of genes related to the PI3K/Akt signaling pathway. The PI3K/Akt pathway has been widely recognized as a key molecular hub in traumatic SCI, exerting anti-apoptotic, pro-axonal regeneration, and anti-inflammatory effects [94, 95]. Therefore, we hypothesize that indole derivatives such as ILA may enhance the transcriptional activity of the PI3K/Akt pathway by activating the upstream AhR signaling axis. Previous studies have demonstrated that AhR can crosstalk with the PI3K/Akt pathway through noncanonical mechanisms in different cell types, mediating the neuroprotective regulation of tryptophan metabolites [96–98]. To verify the central role of AhR in this mechanistic cascade, an AhR inhibitor was administered in addition to BHD-FMT intervention. The results showed that when AhR was inhibited, all beneficial effects of BHD-FMT—including improved motor function, pathological repair of spinal cord and intestinal tissues, and activation of the PI3K/Akt pathway—were substantially attenuated or partially abrogated. This pharmacological evidence suggests that AhR activation contributes to the therapeutic effects of BHD-FMT under the conditions tested. Collectively, AhR, as a receptor for tryptophan metabolites, may exert immunomodulatory and neuroprotective effects by activating the PI3K/Akt signaling pathway, providing key molecular evidence for the therapeutic mechanism of BHD.

By integrating 16S rDNA sequencing, targeted metabolomics, and transcriptomic datasets, this study proposes a systematic “gut–spinal cord axis” therapeutic mechanism. FMT derived from BHD significantly enriched the probiotic *L. johnsonii*, which was strongly correlated with enhanced synthesis of tryptophan-derived metabolites such as ILA. These metabolites, as endogenous agonists of the AhR, can activate the AhR signaling pathway and further modulate the downstream PI3K/Akt pathway, thereby exerting multilevel biological effects, including coordinated anti-inflammatory, neuroprotective, and barrier-repair functions, ultimately promoting neurological recovery after SCI. Collectively, this study elucidates a potential mechanism by which BHD exerts therapeutic effects on SCI through a proposed “*L. johnsonii*–ILA–AhR–PI3K/Akt” regulatory network, thereby deepening the mechanistic understanding of BHD and providing novel therapeutic targets and strategies for SCI intervention. Nevertheless, several limitations should be acknowledged. First, while our multi-omics integration reveals strong correlations within this network, it does not establish definitive causality. The central roles of *L. johnsonii* and ILA, though strongly implicated, require direct validation through loss-of-function (e.g., specific bacterial depletion) and gain-of-function (e.g., ILA supplementation) experiments. Second, although the functional role of the AhR pathway was validated through inhibitor-based experiments, genetic evidence using knockout animal models remains lacking. Third, whether *L. johnsonii* can act as an “candidate probiotic strain” to exert therapeutic effects independently requires further functional validation through mono-strain transplantation experiments. Future studies may focus on establishing causal proof for each node of this axis, developing probiotic consortia, and optimizing the pharmacological properties of ILA-like metabolites to promote precise interventions targeting the gut–spinal cord axis.

## Supplementary Information

Supplementary material 1.

Supplementary material 2.

Supplementary material 3.

Supplementary material 4.

## Data Availability

The data that support the findings of this study are available from the corresponding author, upon reasonable request.

## Abbreviations

AhR: Aryl hydrocarbon receptor
ASVs: Amplicon sequence variants
BHD: Buyang Huanwu Decoction
BBB: Basso, Beattie, Bresnahan
DEGs: Differentially expressed genes
FMT: Fecal microbiota transplantation
H&E: Hematoxylin‒eosin
IAA: Indole-3-acetic acid
ILA: Indole-3-lactic acid
IPA: Indole-3-propionic acid
LC–MS/MS: Liquid chromatography–mass spectrometry
LEfSe: Linear discriminant analysis effect size
*L. johnsonii*: *Lactobacillus johnsonii*
NBD: Neurogenic bowel dysfunction
NF-200: Neurofilament-200
NMDS: Nonmetric multidimensional scaling
PLS-DA: Partial least squares discriminant analysis
RT: Room temperature
SCI: Spinal cord injury
SYN: Synapsin
TCM: Traditional Chinese medicine
TICs: Total ion chromatograms
TJ: Tight junction
VIP: Variable importance in projection
