# Supplementary figures and images for: Buyang Huanwu Decoction promotes neurorepair after spinal cord injury through a Lactobacillus johnsonii–indole-3-lactic acid–AhR–PI3K/Akt axis
Source: Chin Med. 2026 May 8;21:129. doi: 10.1186/s13020-026-01408-x (PMC13154660; doi:10.1186/s13020-026-01408-x)

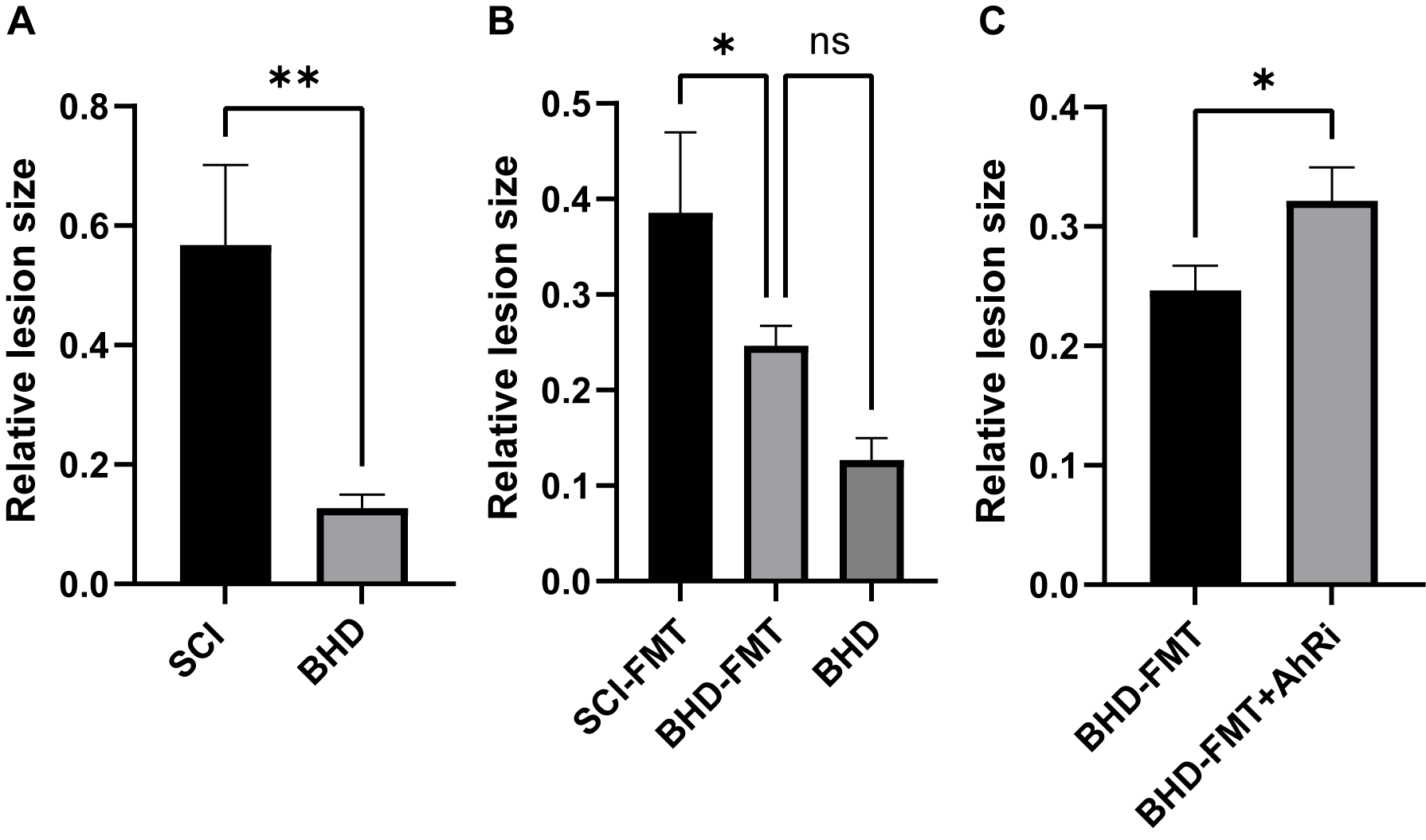

Supplement: Supplementary file 1 — Supplementary material 1. [file 13020_2026_1408_MOESM1_ESM.tif]

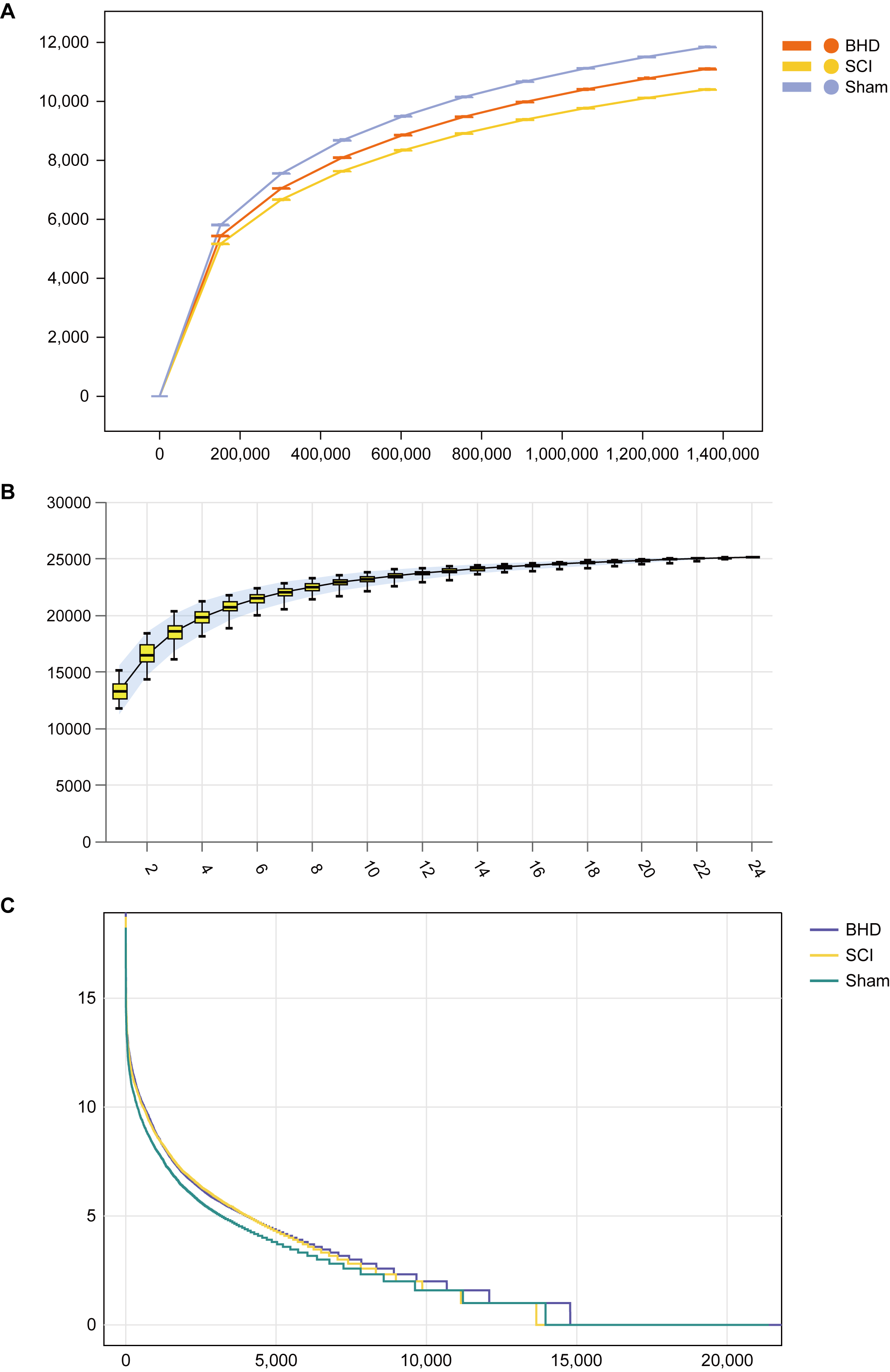

Supplement: Supplementary file 2 — Supplementary material 2. [file 13020_2026_1408_MOESM2_ESM.tif]

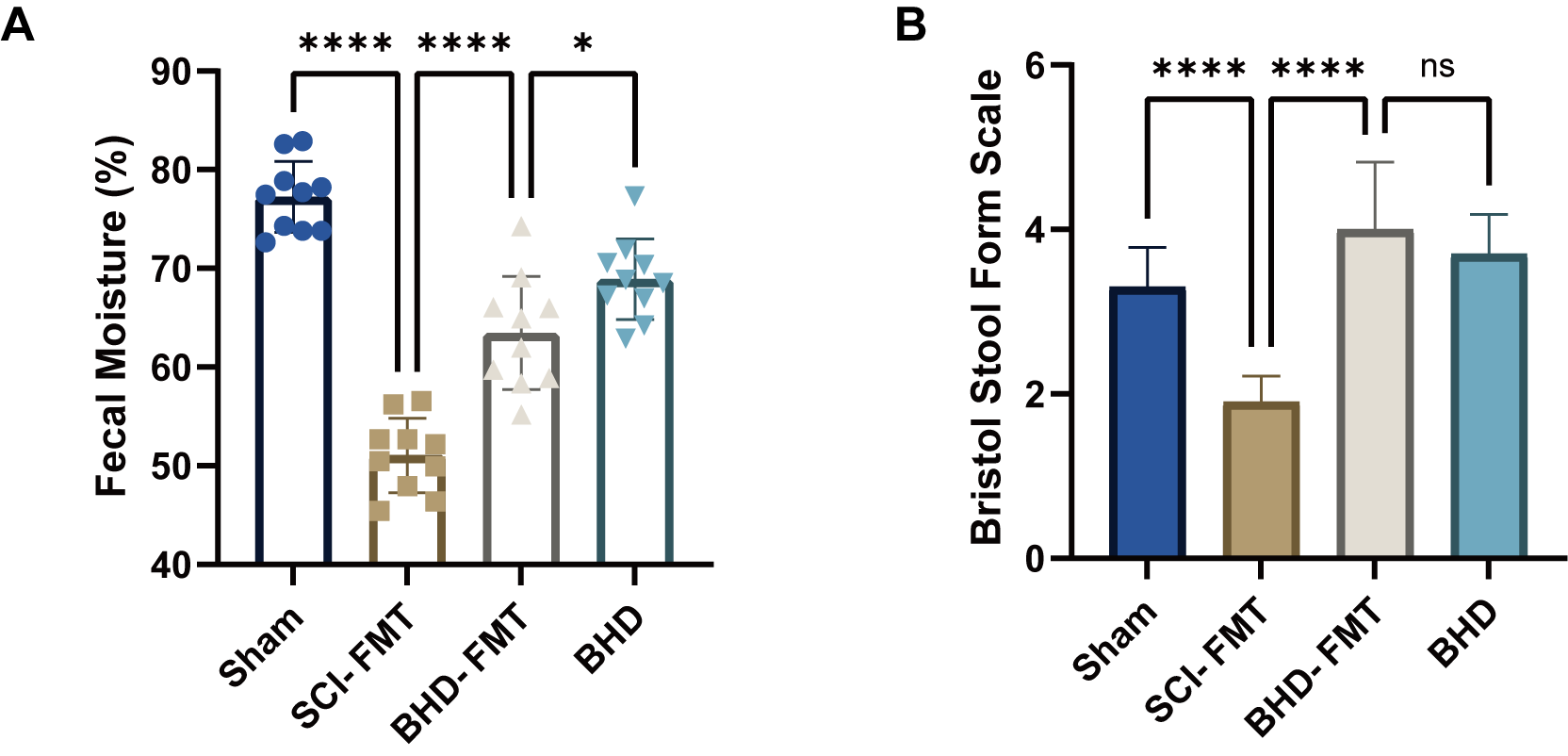

Supplement: Supplementary file 3 — Supplementary material 3. [file 13020_2026_1408_MOESM3_ESM.tif]
