# Supplementary material for: Buyang Huanwu Decoction promotes neurorepair after spinal cord injury through a Lactobacillus johnsonii–indole-3-lactic acid–AhR–PI3K/Akt axis
Source: Chin Med. 2026 May 8;21:129. doi: 10.1186/s13020-026-01408-x (PMC13154660; doi:10.1186/s13020-026-01408-x)
Supplement: Supplementary file 4 — Supplementary material 4. [file 13020_2026_1408_MOESM4_ESM.docx]

**Supplementary Figure 1. Quantitative analysis of spinal cord lesion area from H&E staining.**
**(A)** Quantification of the lesion area (expressed as the ratio of cavity area to total cross-sectional area) in spinal cord tissues from Sham, SCI, and BHD groups. **(B)** Quantification of the lesion area in spinal cord tissues from Sham, SCI-FMT, BHD-FMT, and BHD groups. **(C)** Quantification of the lesion area in spinal cord tissues from Sham, SCI-FMT, BHD-FMT, and BHD-FMT+AhRi groups. **Data are presented as mean ± SD (n = 3 rats per group).** Differences among groups were analyzed by one-way ANOVA followed by Tukey’s post hoc test. **ns**, no significance. *p < 0.05, **p < 0.01, ***p < 0.001, ****p < 0.0001.

**Supplementary Figure 2. Evaluation of sequencing depth and species richness in fecal samples. (A)** Rarefaction curves for each sample group. The curves reaching a plateau indicate that the sequencing depth was sufficient to capture the majority of microbial diversity. **(B)** Species accumulation curve. The curve shows that observed species richness increases with sample size and begins to level off at approximately 24 samples, suggesting adequate sampling effort. **(C)** Abundance rank curve. The distribution reflects the richness (number of species) and evenness (relative abundance distribution) of the microbial community within each sample group.

**Supplementary Figure 3. Quantitative assessment of intestinal function at day 21 post-SCI. (A)** Fecal water content in the indicated groups. **(B)** Bristol Stool Form Scale (BSFS) scores in the indicated groups. **Data are presented as mean ± SD (n = 10).** Differences among groups were analyzed by one-way ANOVA followed by Tukey’s post hoc test. **ns**, no significance. *p < 0.05, **p < 0.01, ***p < 0.001, ****p < 0.0001.

**Supplementary Table 1. Primer sequences used for qPCR**

| **Primer name** | **Forward sequence (5'→3')** | **Reverse sequence (5'→3')** |
| --- | --- | --- |
| Il1b | TCTCACAGCAGCATCTCGACAAG | CCACGGGCAAGACATAGGTAGC |
| Il6 | GAGACTTCCAGCCAGTTGCCTTC | CTGGTCTGTTGTGGGTGGTATCC |
| Cd68 | ACTTGGCTCTCTCATTCCCTTACG | GGTAGACTGTACTGTGGCTCTGATG |
| Cd206 | CTTGACCTCTGGACTCTGGATTGG | CTGATGATGGACTTCCTGGTAGCC |
| Gapdh | CAAGTTCAACGGCACAGTCAAGG | ACATACTCAGCACCAGCATCACC |

**Supplementary Table 2. Identified tryptophan metabolites in fecal samples after BHD-FMT treatment.**

| **Index** | **Compounds** | **Class** | **Ion mode** | **Ionization model** | **Formula** |
| --- | --- | --- | --- | --- | --- |
| 2-AF | 2-Aminophenol | Aniline Compounds | Positive | [M+H]+ | C6H7NO |
| N-Acid | Nicotinic acid | Pyridines and derivatives | Positive | [M+H]+ | C6H5NO2 |
| PA | Picolinic acid | Pyridines and derivatives | Positive | [M+H]+ | C6H5NO2 |
| TRM | Tryptamine | Indoles and derivatives | Positive | [M+H]+ | C10H12N2 |
| NAS | N-acetylserotonin | Indoles and derivatives | Positive | [M+H]+ | C12H14N2O2 |
| IDA | 3-Indoleacrylic acid | Indoles and derivatives | Negative | [M-H]- | C11H9NO2 |
| SER | Serotonin | Indoles and derivatives | Positive | [M+H]+ | C10H12N2O |
| L-TRP | L-tryptophan | Indoles and derivatives | Positive | [M+H]+ | C11H12N2O2 |
| KYNA | Kynurenic acid | Quinolines and derivatives | Negative | [M-H]- | C10H7NO3 |
| IPA | Indolylpropionic acid | Indoles and derivatives | Positive | [M+H]+ | C11H11NO2 |
| NFK | N-formylkynurenine | Carbonyl compounds | Positive | [M+H]+ | C11H12N2O4 |
| L-KYN | L-kynurenine | Carbonyl compounds | Positive | [M+H]+ | C10H12N2O3 |
| NFAA | N-Formylanthranilic acid | Benzoic acids and derivatives | Negative | [M-H]- | C8H7NO3 |
| 2-AA | 2-Aminobenzoic acid | Benzoic acids and derivatives | Positive | [M+H]+ | C7H7NO2 |
| ICAld | Indole-3-carbaldehyde | Indoles and derivatives | Positive | [M+H]+ | C9H7NO |
| IAA | β-Indole-3-acetic acid | Indoles and derivatives | Positive | [M+H]+ | C10H9NO2 |
| 3-HKYN | 3-Hydroxykynurenine | Carbonyl compounds | Positive | [M+H]+ | C10H12N2O4 |
| IEt | Tryptophol | Indoles and derivatives | Positive | [M+H]+ | C10H11NO |
| XA | Xanthurenic acid | Quinolines and derivatives | Negative | [M-H]- | C10H7NO4 |
| 5-HTOL | 5-Hydroxytryptophol | Indoles and derivatives | Positive | [M+H]+ | C10H11NO2 |
| CA | Cinnabarinic acid | Oxazines | Negative | [M-H]- | C14H8N2O6 |
| ILA | Indole-3-lactic acid | Indoles and derivatives | Negative | [M-H]- | C11H11NO3 |
| 2-KA | 2-Ketoadipic acid | Keto acids and derivatives | Negative | [M-H]- | C6H8O5 |
| 5-HTP | Hydroxytryptophan | Indoles and derivatives | Positive | [M+H]+ | C11H12N2O3 |
| IAM | Indole-3-acetamide | Indoles and derivatives | Negative | [M-H]- | C10H10N2O |
| 3-IS | Indoxylsulfate | Indoles and derivatives | Negative | [M-H]- | C8H7NO4S |
| IACN | 3-Indoleacetonitrile | Indoles and derivatives | Positive | [M+H]+ | C10H8N2 |
| 5-HIAA | 5-Hydroxyindoleacetic acid | Indoles and derivatives | Positive | [M+H]+ | C10H9NO3 |
| 5-MeOT | 5-Methoxytryptamine | Indoles and derivatives | Positive | [M+H]+ | C11H14N2O |
| IGA | 3-Indoleglyoxylic acid | Indoles and derivatives | Positive | [M+H]+ | C10H7NO3 |
| MTRM | N-Methyltryptamine | Indoles and derivatives | Positive | [M+H]+ | C11H14N2 |
| 3-HAA | 3-Hydroxyanthranilic acid | Benzoic acids and derivatives | Negative | [M-H]- | C7H7NO3 |
| 5-Me-IAA | 5-Methoxyindoleacetic acid | Indoles and derivatives | Positive | [M+H]+ | C11H11NO3 |
